# Impaired gephyrin G-domain trimerization and phase separation in a patient with developmental epileptic encephalopathy

**DOI:** 10.1038/s44321-026-00474-w

**Published:** 2026-06-30

**Authors:** Emanuel H W Bruckisch, Marcelo de Melo Aragão, Thais dos Santos Rohde, Ann-Kathrin Huber, Mateus de Oliveira Torres, Günter Schwarz, Filip Liebsch

**Affiliations:** 1 Institute of Biochemistry, Department of Chemistry, University of Cologne, 50674 Cologne, Germany; 2 Department of Neurology & Neurosurgery, University Hospital São Paulo, Federal University of São Paulo (UNIFESP), São Paulo, Brazil; 3 Cologne Excellence Cluster on Cellular Stress Responses in Aging-Associated Diseases (CECAD), University of Cologne, Cologne, Germany; 4 Center for Molecular Medicine Cologne (CMMC), University of Cologne, Cologne, Germany

**Keywords:** Genetics, Gene Therapy & Genetic Disease, Neuroscience

## Abstract

Epilepsy, a common neurological disorder is frequently linked to genetic variants in synaptic proteins. Here, we describe a de novo pathogenic missense variant in the gephyrin G-domain (G134R) identified in an individual with developmental delay, epileptic seizures, microcephaly, dysmorphic features and short stature. Functional analyses reveal that G134R disrupts higher-order oligomerization, leading to impaired liquid–liquid phase separation (LLPS) and synaptic clustering. Recombinant G134R-gephyrin variant forms lower oligomers and retains only 60% of its molybdenum cofactor (Moco) synthesis activity while binding to glycine receptor models is unaffected. In non-neuronal cells, G134R fails to oligomerize beyond dimers with Moco synthesis activity reduced to 5%. In neurons, G134R is unable to form synaptic clusters and exerts a dominant-negative effect on WT-gephyrin, severely disrupting inhibitory synapse formation. Our findings highlight a critical role for the G-domain in gephyrin self-assembly and LLPS, shifting the focus from the E-domain-centric view of gephyrin function and providing a novel molecular mechanism for epilepsy linked to G-domain mutations.

## Synopsis

A de novo gephyrin G-domain mutation causing epilepsy and developmental delay is shown to abolish higher-order oligomerization and liquid–liquid phase separation, revealing a dominant-negative effect at inhibitory synapses and reframing the molecular basis of gephyrin-linked neurological disease.

- A novel de novo missense variant (G134R) in the gephyrin G-domain is identified in a patient presenting with epileptic seizures, developmental delay, microcephaly, dysmorphic features, and short stature.
- Higher-order oligomerization and liquid–liquid phase separation (LLPS) of gephyrin are shown to be critically dependent on G-domain integrity, independent of glycine receptor binding.
- Molybdenum cofactor (Moco) biosynthesis activity is reduced to ~60% in recombinant G134R-gephyrin and to ~5% in non-neuronal cells, while receptor-binding affinity is found to be unaffected.
- Synaptic clustering of gephyrin is abolished in neurons expressing G134R, and a dominant-negative effect on wild-type gephyrin is demonstrated, severely impairing inhibitory synapse formation.

## Abstract (web summary)

A de novo gephyrin G-domain mutation causing epilepsy and developmental delay is shown to abolish higher-order oligomerization and liquid–liquid phase separation, revealing a dominant-negative effect at inhibitory synapses and reframing the molecular basis of gephyrin-linked neurological disease.

## Graphical abstract

**Figure:**
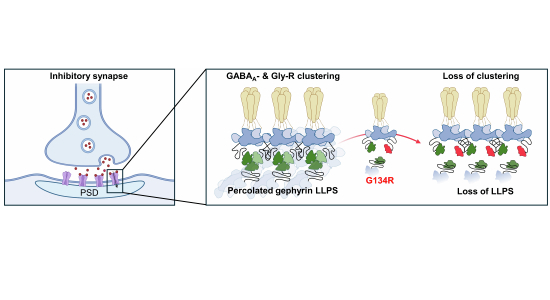

**Figure:**
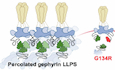

The paper explained **Problem**

The brain relies on a careful balance between excitatory and inhibitory signals to function normally. When this balance is disrupted, for example because inhibitory synapses fail to form properly, this can result in epilepsy, one of the most common neurological disorders worldwide. Many epilepsy cases are now known to arise from spontaneous mutations in single genes, but the precise molecular mechanisms often remain poorly understood, making diagnosis and treatment development difficult.

**Results**

In this study, we report a child born with epileptic seizures and developmental delay, caused by a single change in the gene encoding gephyrin, a key scaffolding protein that anchors inhibitory receptors at neuronal contacts. We show that this mutation (G134R) sits in a part of gephyrin called the G-domain and prevents the protein from assembling into the large molecular clusters it normally forms. Without this self-assembly, gephyrin cannot undergo the droplet-like condensation process that concentrates it at inhibitory synapses. In neurons, the mutant protein not only fails to do its job, but actively interferes with the healthy copy of gephyrin still present in the cell, a so-called dominant-negative effect, causing a near-complete breakdown of inhibitory synapse formation.

**Impact**

These findings reframe how scientists and clinicians think about gephyrin-related disease, shifting attention to a part of the protein that was previously underappreciated. They explain why a single inherited mutation is sufficient to cause severe neurological symptoms and will aid the interpretation of newly discovered gephyrin variants in epilepsy diagnostic panels. Longer term, restoring gephyrin self-assembly emerges as a plausible therapeutic strategy for this and related forms of genetic epilepsy.

## Introduction

Epilepsy, affecting over 50 million people worldwide, is a common neurological disorder characterized by recurrent seizures caused by an imbalance between excitatory and inhibitory neuronal activity (Schwartzkroin, 2012; Yardi, 2025). Among the most severe forms are developmental and epileptic encephalopathies (DEE) (Scheffer et al, 2025), which often arise from de novo genetic mutations and manifest in early childhood with seizures and are regularly accompanied by developmental delay, and cognitive impairment (Cross and Guerrini, 2013). Mutations in genes involved in synaptic transmission are frequent causes of DEE, with inhibitory synaptic proteins playing a prominent role (Guerrini et al, 2023; Uzay and Kavalali, 2023; Reinthaler et al, 2015).

Gephyrin is the essential postsynaptic scaffold protein at inhibitory synapses. It interacts with glycine receptors (GlyRs) (Meyer et al, 1995) and GABA type A receptors (GABA_A_Rs) (Maric et al, 2011), thereby anchoring and clustering these receptors to maintain inhibitory strength (Fritschy et al, 2008; Tyagarajan and Fritschy, 2014). Gephyrin assembles into higher-order oligomeric complexes, mediated by trimerization of its N-terminal G-domain (Schwarz et al, 2001; Saiyed et al, 2007) and dimerization of the C-terminal E-domain (Kim et al, 2006). Recently, gephyrin oligomerization has been linked to liquid–liquid phase separation (LLPS), proposed as a central mechanism for the formation and plasticity of inhibitory synapses (Bai et al, 2021; Bai and Zhang, 2022; Zhu et al, 2024; Lee et al, 2024).

**Figure 1:**
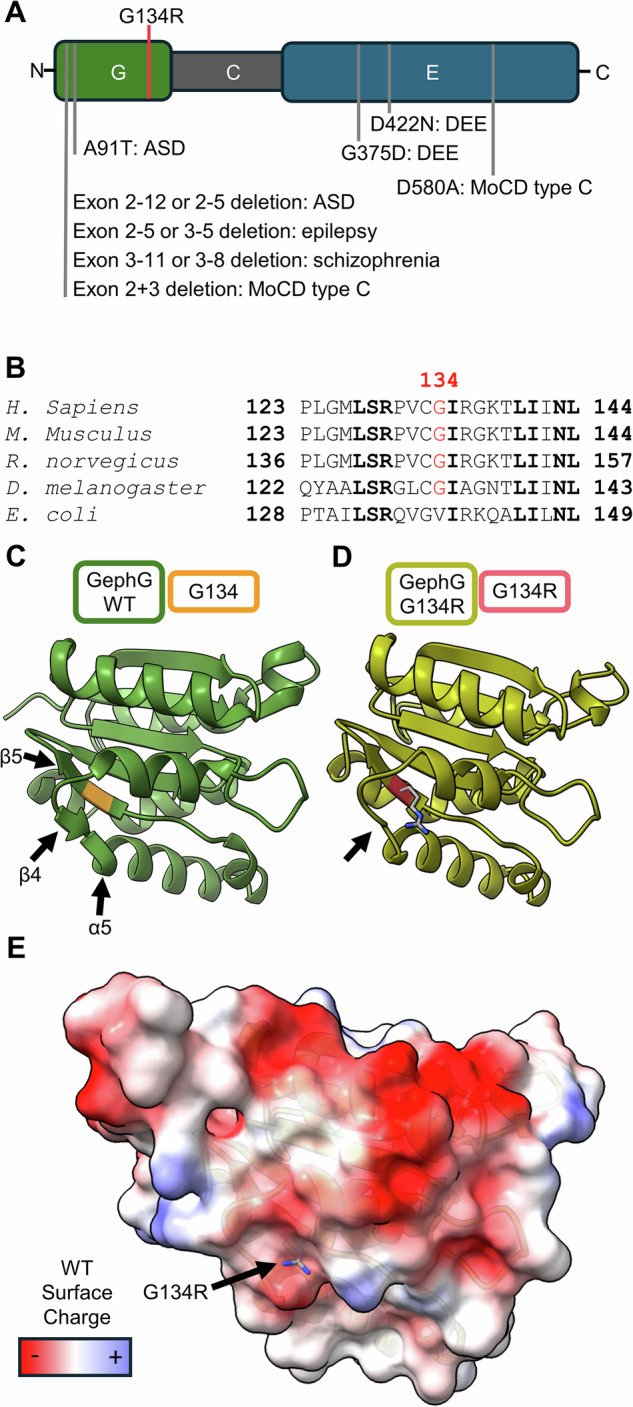
Localization of the identified *GPHN* mutation G134R. (**A**) Gephyrin domain structure with localization of G134R. (**B**) Local sequence alignment of homologous G-domains in different species. Amino acids in bold are conserved. (**C**) X-ray crystal structure of human gephyrin G-domain monomer (PDB: 1JLJ). Yellow marks glycine 134. Arrows indicate alpha-helix 5 (α5) which is important for trimerization, beta-sheet 4 (β4) and beta-sheet 5 (β5) which neighbor alpha-helix 5. (**D**) AlphaFold3 prediction of G134R-gephyrin G-domain monomer. Magenta marks the position of G134R with arginine’s carbon atoms depicted in gray and nitrogen in blue. Arrow marks the position of the lost beta-sheet 4 (β4). (**E**) Electrostatic surface prediction taken from (**C**) overlayed onto (**D**) with arrow marking G134R. Red indicates negative charge, blue positive charge. The positive arginine side chain protrudes into a net negative surface charge. Source data are available online for this figure.

Besides its synaptic role, gephyrin catalyzes the final steps of molybdenum cofactor (Moco) biosynthesis, a function conserved from bacteria to humans (Belaidi and Schwarz, 2013). In this pathway, the G-domain catalyzes the adenylation of molybdopterin (MPT) to generate MPT–AMP, while the E-domain subsequently hydrolyzes AMP and incorporates molybdate to yield active Moco (Belaidi and Schwarz, 2013). Mutations in gephyrin can thus impair metabolic functions, and a homozygous deletion of exons 2+3 in the gephyrin gene (*GPHN*) resulted in complete loss of gephyrin protein and caused molybdenum cofactor deficiency (MoCD) type C (Fig. 1A) (Schwarz et al, 2009; Johannes et al, 2022; Reiss et al, 2001).

Pathogenic variants and exonic microdeletions in *GPHN* have been identified in patients with a spectrum of neurodevelopmental and neuropsychiatric disorders, including autism spectrum disorder (ASD), schizophrenia, and epilepsy (Fig. 1A) (Lionel et al, 2013). Mechanistic studies on patients demonstrated that missense variants within the E-domain, such as G375D and D422N reduce gephyrin receptor-binding and clustering, thereby disrupting inhibitory neurotransmission and causing DEE (Fig. 1A) (Macha et al, 2022; Dejanovic et al, 2015). Additionally, single particle cryogenic electron microscopy analyses revealed that these two variants fail to form filaments via subdomain II interactions of adjacent dimers, which likely explains their inability to form higher oligomers and undergo LLPS (Bai et al, 2021; Kim et al, 2021; Macha et al, 2025).

Here, we describe the first pathogenic gephyrin patient variant with a single amino acid exchange located in the G-domain, derived from an epileptic patient. We identified a heterozygous de novo missense mutation (c.400G>A, p.Gly134Arg “G134R”) in a patient with developmental seizures born to unrelated and unaffected parents. Functional analyses revealed that G134R disrupted the ability of gephyrin to form higher-order oligomers. We hypothesize that while the E-domain serves as the primary driver of phase separation and provides the interface for receptor binding (Macha et al, 2025), the G-domain plays a crucial role in interconnecting gephyrin conjugates into higher-order oligomers, thereby enhancing the capacity for phase separation and supporting synaptic clustering. Consequently, G134R-gephyrin failed to generate functional receptor-scaffold complexes at inhibitory synapses and similar to G375D-gephyrin decreased the density of synaptic WT clusters in a dominant-negative fashion.

## Results

### Clinical description of the patient with a de novo G134R-gephyrin variant

We report a 15-year-old female patient from Brazil, born late preterm after an uneventful pregnancy and delivery. No prenatal, perinatal, or postnatal complications were reported. Her parents are unrelated, and there is no family history of epilepsy or other neurological disorders. Developmental delay was noted within the first year of life, and at the age of 9 months, she was diagnosed with epilepsy. Seizures occurred predominantly during sleep and were characterized by apnea, generalized hypotonia, and episodes of tonic posturing of all four limbs followed by clonic movements lasting several minutes. During childhood, she was further diagnosed with intellectual disability and short stature.

Clinical follow-up began when the patient was 10 years old. Neurological examination at that time showed microcephaly and minor dysmorphic features, including bilateral epicanthus, upslanting almond-shaped palpebral fissures, and small, low-set ears with attached earlobes. Brain MRI performed at 5 years of age revealed no structural abnormalities. Electroencephalography (EEG) at 10 years demonstrated epileptiform discharges, predominantly in the left temporal region during drowsiness. It is likely that the patient’s epileptic events began during infancy, at approximately nine months of age. During this period, the semiology consisted of brief episodes of apnea and hypotonia lasting a few seconds, consistently occurring during sleep. Between the ages of 13 years and 7 months and 13 years and 11 months, prior to the initiation of treatment with the benzodiazepine clobazam, she exhibited a seizure frequency of approximately one episode per month. These events were characterized by tonic posturing of all four limbs accompanied by staring, occurred exclusively during sleep, and lasted several minutes. Before starting clobazam treatment, the patient had undergone therapeutic trials with topiramate, valproate, lamotrigine, and levetiracetam. However, adherence was consistently poor, as she did not tolerate or adequately accept these medications. Clobazam treatment was initiated at the age of 14 years and 3 months, at a dose of 5 mg, and she remained on this regimen thereafter. At the age of 15 years and 1 month, she experienced a single brief breakthrough seizure, prompting an increase in the dose to 10 mg at the age of 15 years and 3 months, approximately one year after treatment initiation. Since the dosage adjustment, she has maintained good seizure control, with no further reported events.

Genetic testing was performed to further investigate the cause of her condition. Both karyotyping and chromosomal microarray analysis yielded normal results. Subsequent next-generation sequencing (NGS by Invitae Corporation, San Francisco, USA) of a gene panel for epilepsy and metabolic disorders identified a heterozygous missense variant in *GPHN*; (NM_001024218.2: c.400G>A; p.Gly134Arg). Although an additional intronic variant in *SCN9A* (encoding the sodium voltage-gated channel alpha subunit 9) was identified, it was only classified as “likely benign”, already detected in the unaffected mother, and is primarily associated with altered pain perception rather than epilepsy (Ghanty et al, 2025; Fertleman et al, 2006); therefore, we focused our analyses on the gephyrin variant. This gephyrin variant was initially classified as a variant of uncertain significance (VUS). Segregation analysis demonstrated that the variant had arisen de novo. The affected residue Gly134 is located within the G-domain of gephyrin (Fig. 1A) and is conserved throughout higher eukaryotes (Fig. 1B) suggesting an important structural role. Looking at the published crystal structure it becomes obvious that Gly134 resides within the β-sheet 5 structure adjacent to α-helix 5, which forms the trimerization interface (Fig. 1C; PDB 1JLJ). In silico prediction tools uniformly support a deleterious effect of the Gly-to-Arg exchange: PolyPhen-2 predicts the substitution to be “probably damaging” (score 1.000), SIFT predicts it to “affect protein function” (score 0.01), MutationTaster25 classifies the variant as “deleterious” and the combined annotation dependent depletion (CADD) score lists it in the top 0.05% most deleterious potential substitutions across the human genome (score 33.0). Furthermore, the human genome variant database “GnomAD” does not list any allele frequency for this variant, underlining the extreme rarity (variant ID: 14-66916013-G-A). AlphaFold3-based structural prediction suggests that the G134R substitution perturbs the β-sheet 4 element (Fig. 1D), with the bulky, positively charged arginine side chain protruding from and disrupting the otherwise electronegative surface defined by the WT crystal structure (Fig. 1E). Additionally, to complement the AlphaFold3 prediction, we used the web server DDMut-PPI (prediction of mutation-induced changes in protein–protein interaction) to calculate the predicted change in binding free energy of G-domain trimerization (ΔΔGBinding), which yielded a decreased affinity value (−2.53 ± 0.3 kcal/mol). Based on these findings, we decided to further study the molecular and functional consequences of G134R-gephyrin in vitro and in cells.

### Impaired gephyrin self-assembly and structural destabilization by G134R

**Figure 2:**
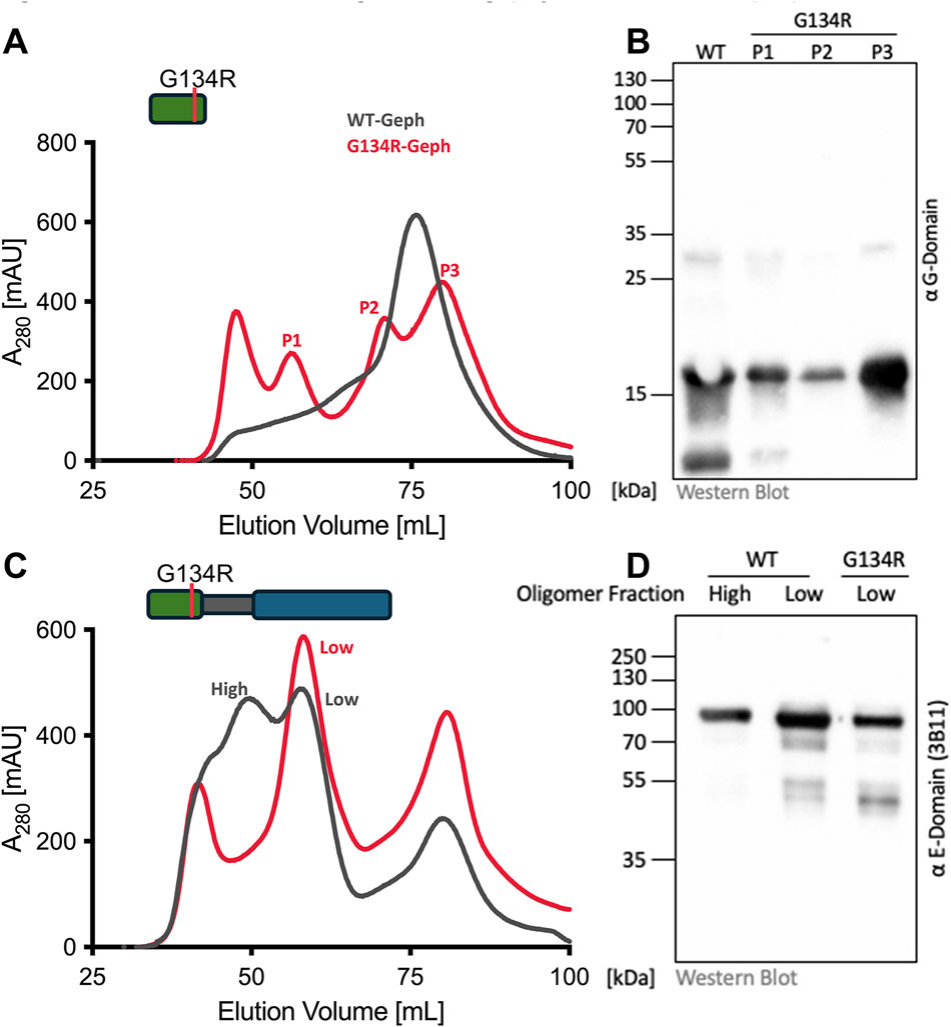
G-domain and full-length G134R-gephyrin show distinct properties in vitro. (**A**) Size exclusion chromatography (SEC) elution profile of G-domain WT-gephyrin (grey) and G134R-gephyrin (magenta). While WT-gephyrin shows distinct trimeric elution peak, G134R-gephyrin splits up into 4 peaks. (**B**) Western blot analysis of eluted fractions from (**A**) identifies G134R-gephyrin peaks P1-3 via G-domain specific antibody. The column void volume is ~40 mL. (**C**) SEC elution profile of full-length WT-gephyrin (grey) and G134R-gephyrin (magenta). While WT-gephyrin shows elution peaks for trimeric low-oligomerization gephyrin and an additional peak for high-oligomerization, G134R-gephyrin is missing the latter. The column void volume is ~40 mL and gephyrin degradation products are detected at ~80 mL. (**D**) Western blot analysis of eluted fractions from (**C**) identifies G134R-gephyrin via E-domain specific antibody (3B11). Only low-oligomer fractions show characteristic double band behavior. Source data are available online for this figure.

To investigate the biochemical properties of G134R-gephyrin, we expressed and purified recombinant isolated G-domain variants and full-length gephyrin from *E. coli*. Size-exclusion chromatography (SEC) of the purified G-domain confirmed the expected trimeric assembly for WT gephyrin, yielding a single, well-defined peak at 75 mL elution volume (Fig. 2A). In contrast, the G134R G-domain did not elute as a single trimer peak but produced partially overlapping peaks surrounding the elution of the WT peak. All fractions were positive for the G-domain as detected by Western blot analysis, indicating that the protein variant is present in multiple distinct oligomeric states (Fig. 2B). These findings suggest that the canonical trimerization of the G-domain is altered by the G134R substitution.

Analysis of full-length gephyrin revealed the characteristic oligomerization elution-profile previously described for WT protein, with specific populations corresponding to lower trimeric and higher oligomeric assemblies (50 mL and 60 mL, respectively, in Fig. 2C), in addition to aggregates eluting at 40 mL and degradation products eluting at 80 mL. Strikingly, full-length G134R-gephyrin completely lacked the high-oligomer peak at 50 mL, while only the low trimeric fraction was detectable. These trimers eluted at 60 mL, migrated at ~85 kDa on SDS–PAGE, and were indistinguishable from WT gephyrin by Western blot analysis, indicating that these units remain intact but fail to engage in higher-order assembly (Fig. 2D).

Given these profound alterations in the oligomerization properties of G134R-gephyrin, we next asked whether the protein retains its biological functions. We therefore performed in-depth biochemical analyses of the two major activities of gephyrin: clustering of inhibitory neurotransmitter receptors and catalysis of the molybdenum cofactor (Moco) biosynthesis.

### G134R-gephyrin showed unaffected receptor binding and reduced but functional Moco synthesis activity

**Figure 3:**
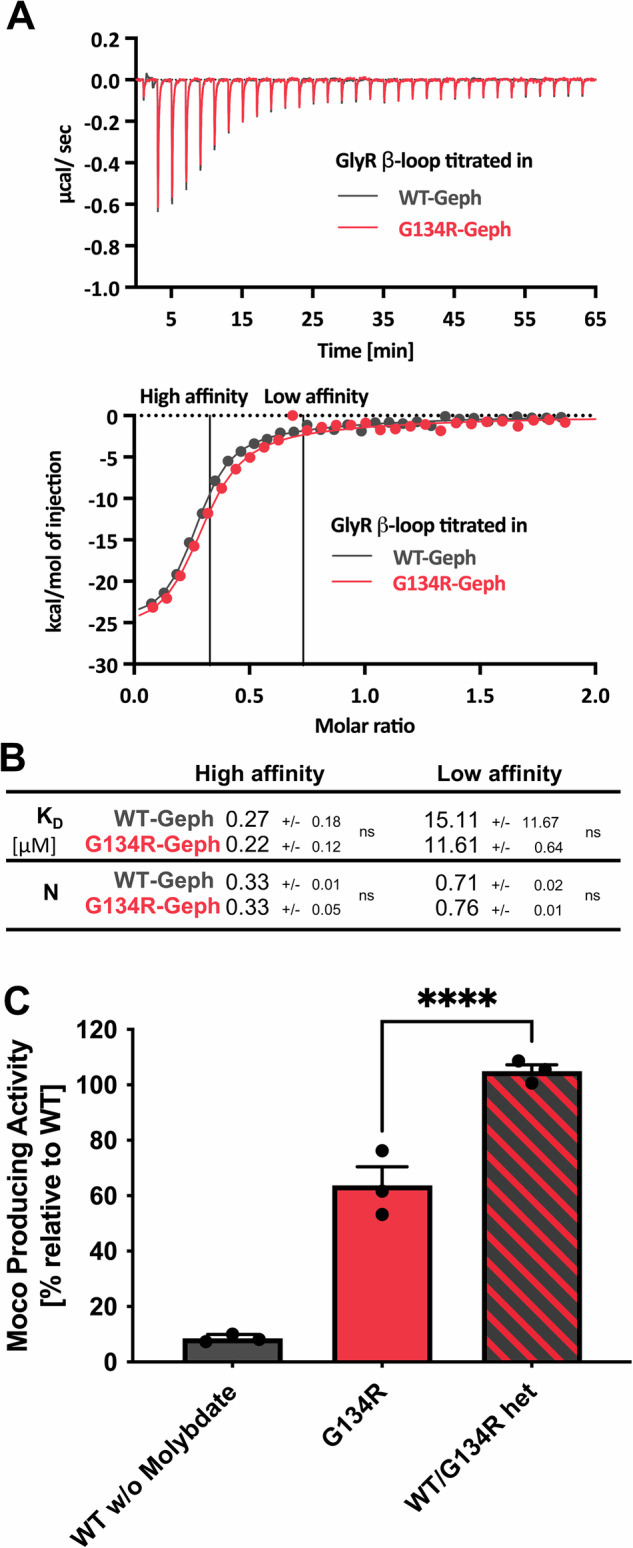
Preserved GlyR β-Loop binding and Moco activity in G134R-gephyrin in vitro. (**A**) Representative isothermal titration calorimetry (ITC) binding curves illustrating the interaction of the GlyR β-loop with either WT-gephyrin or G134R-gephyrin. Experimental data were fitted with a two-site binding model (solid lines), distinguishing high- and low-affinity interaction sites. (**B**) Quantitative binding parameters obtained from the ITC measurements, including dissociation constants (KD, μM) and stoichiometry values (N), are presented. Data are reported as mean ± SD (*n* = 3, derived from three independent protein purifications) and statistical comparisons were performed using an unpaired, two-tailed Student’s t-test. No significant differences were detected for any of the parameters (ns, *p* ≥ 0.05). (**C**) In vitro molybdenum cofactor (Moco) biosynthesis assay was performed using 150 pmol of purified 6His-tagged gephyrin proteins as described earlier (Belaidi and Schwarz, 2013). Presented as % relative to WT-gephyrin activity. Reaction lacking molybdenum (w/o molybdate) were included as internal negative control to validate assay performance. Data are reported as mean ± SD (*n* = 3, derived from three independent measurements) and statistical comparisons were performed using one-way ANOVA (F(4,10) = 170.5, *p* = 3.74 × 10^−9^) with Tukey’s multiple comparisons test (p_G134R vs WT/G134R het_ = 0.00018; ****). Source data are available online for this figure.

E-domain dimers of gephyrin mediate the postsynaptic anchoring of inhibitory receptors by binding to conserved intracellular loop domains of GlyR β- and GABA_A_R α3-subunits. Although Gly134 is located distantly in the N-terminal G-domain, the loss of higher-order oligomerization in G134R-gephyrin prompted us to test whether this variant affects receptor binding. To this end, we performed isothermal titration calorimetry (ITC) experiments using a synthetic peptide derived from the GlyR β intracellular loop domain (GlyR β-ICD). As previously reported (Macha et al, 2022), this peptide is a suitable model to study gephyrin–receptor interactions since GlyRs and GABA_A_Rs compete for the same binding site on gephyrin (Maric et al, 2014). In agreement with earlier studies, WT-gephyrin showed two distinct binding sites, a high-affinity interaction in the sub-micromolar range (K_*D*_ = 0.27 µM, N = 0.33) and a low-affinity interaction in the double-digit micromolar range (K_*D*_ = 15.11 µM, N = 0.71) (Fig. 3B). Strikingly, the G134R variant displayed binding curves and thermodynamic parameters highly similar to those of WT protein (Fig. 3A), with no statistically significant differences in affinity or stoichiometry (Fig. 3B). These results indicate that, despite its profound oligomerization defects, G134R-gephyrin retains intact receptor-binding capacity in vitro.

Gephyrin is a moonlighting protein with a second essential function in cellular metabolism: catalysis of the final two steps in Moco biosynthesis. We therefore assessed whether structural alterations in the G-domain of G134R-gephyrin impair its enzymatic activity. In vitro Moco synthesis assays revealed that G134R-gephyrin variant produced ~400 pmol Moco, corresponding to 64 ± 9% of WT activity (Fig. 3C). Thus, although the G134R substitution markedly reduces catalytic efficiency, it does not completely abolish enzymatic activity. Importantly, when WT and G134R proteins were mixed in a 1:1 ratio mimicking the heterozygous expression condition in the patient, Moco production was restored to ~700 pmol, corresponding to 105 ± 3% of WT activity (Fig. 3C). These findings suggest that G134R-gephyrin does not exert a dominant-negative effect on the enzymatic function of WT-gephyrin.

Taken together, our results demonstrate that G134R-gephyrin preserves its two core functions in vitro: receptor binding and Moco synthesis. The partial Moco activity is likely mediated by the trimers that still form in vitro and are presumed to remain catalytically competent. Consistent with the absence of structural brain abnormalities on MRI and with the lack of biochemical or clinical signs of Moco deficiency, this is not expected to be clinically relevant. Instead, our data point toward defective higher-order self-assembly as the critical impairment of this variant. Given the established link between gephyrin receptor clustering and LLPS at inhibitory synapses, we next investigated whether the inability of G134R-gephyrin to form higher-order assemblies translates into impaired phase separation capacity.

### Loss of higher-order oligomers impairs gephyrin phase separation

**Figure 4:**
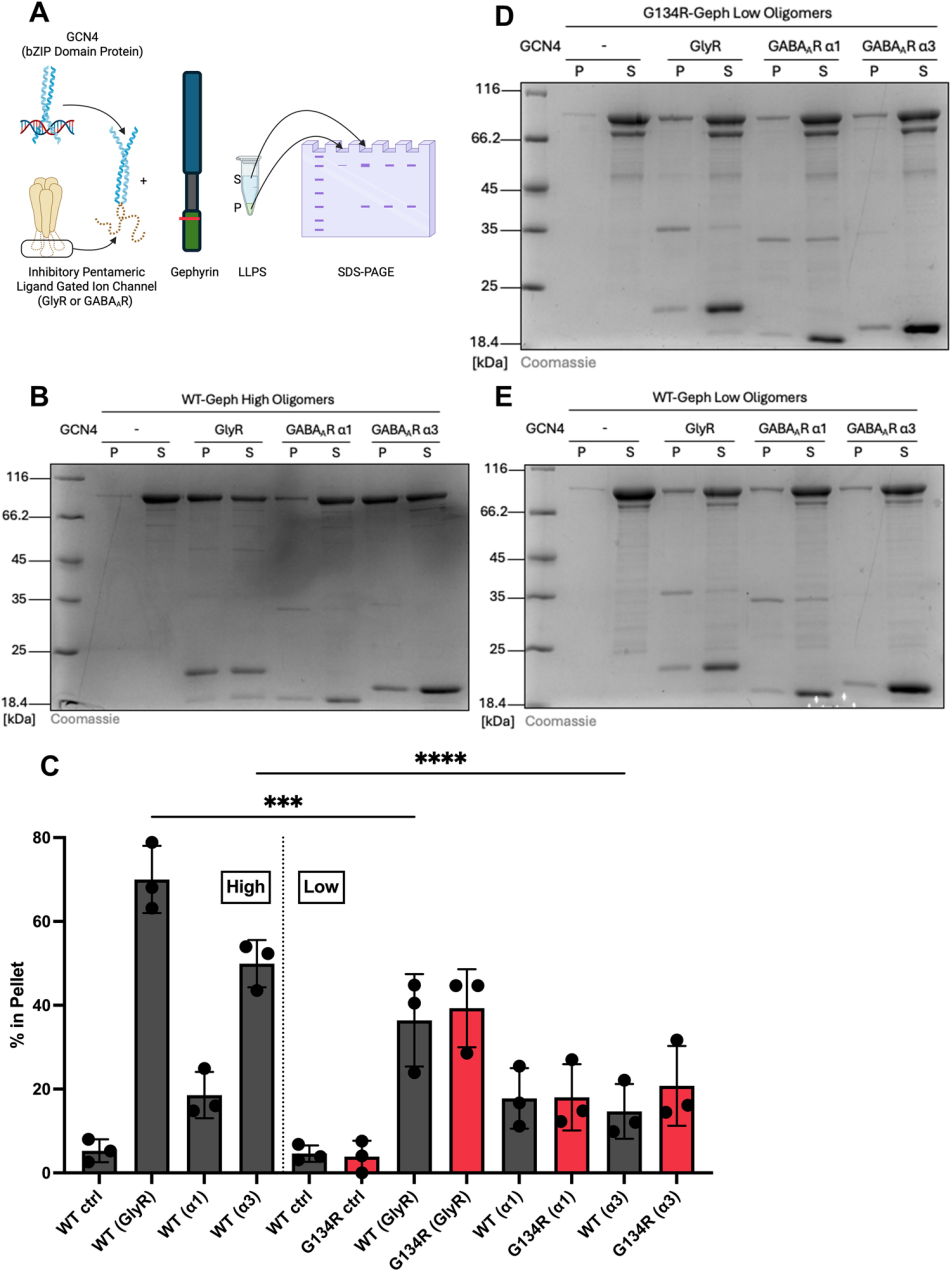
A loss of higher oligomers reduces G134R gephyrin LLPS in vitro. (**A**) Cartoon depicting GCN4-based receptor models (Bai et al, 2021) and experimental design of liquid–liquid phase separation (LLPS) sedimentation assay. (**B**) Representative Coomassie stained SDS-PAGE of LLPS sedimentation assay from WT-gephyrin high oligomers with GCN4-based receptor models for GlyR, GABA_A_R-α1, and GABA_A_R-α3 showing the amount of protein present in pellet (P) and supernatant (S) fractions. (**C**) The band intensities in the pellet fraction were measured and are shown as mean ± SD from three independent experiments (*n* = 3) and statistical comparisons were performed using a two-way ANOVA (F(15,22) = 25.69, *p* = 2.39 × 10^−10^) with Tukey’s multiple comparisons test (p_WToligomer vs WTtrimer_ (GlyR) = 0.000123 (***), p_WToligomer vs WTtrimer_ (α3) = 0.000061 (****)). (**D**,** E**) Representative Coomassie stained SDS-PAGE of LLPS sedimentation assay from G134R-gephyrin low oligomers (**D**) and WT-gephyrin low oligomers (**E**) with GCN4-based receptor models for GlyR, GABA_A_R-α1 and GABA_A_R-α3 showing the amount of protein present in pellet (P) and supernatant (S) fractions. Source data are available online for this figure.

Given that gephyrin synaptic clustering has been linked to LLPS at inhibitory synapses, we next tested whether the inability of gephyrin-G134R to form higher oligomers alters its phase separation behavior. We employed an established assay (Bai et al, 2021), in which the ICDs of inhibitory receptors are fused to the dimeric yeast zinc finger protein GCN4 and used them to probe receptor-induced gephyrin phase separation in sedimentation experiments (Fig. 4A). As described previously, the GlyR β-ICD represents the strongest known gephyrin interactor, the GABA_A_R α3-ICD shows intermediate affinity, and the GABA_A_R α1-ICD interacts only weakly.

When tested with the high oligomeric fraction of WT-gephyrin (isolated after SEC purification), we observed robust receptor-specific phase separation. Within the sedimented pellet fraction, 70 ± 7% of gephyrin were recovered when GlyR β-ICD fusion protein was present, while 50 ± 5% sedimented with GABA_A_R α3-ICD, and 19 ± 5% with GABA_A_R α1-ICD, consistent with the expected order of interaction strengths (Fig. 4B,C “High”). In contrast, the lower trimeric fraction of WT gephyrin showed significantly reduced phase separation with GlyR β-ICD (*p* = 0.000123) and α3-ICD (*p* = 0.000061) pelleting decreased to 36 ± 9% and 15 ± 5%, respectively, while α1-ICD interactions remained unchanged (Fig. 4E,C). These results highlight the critical contribution of higher-order oligomers for phase separation. G134R-gephyrin, which lacks the ability to form higher oligomers, behaved similarly to WT trimers in LLPS (Fig. 4D,C “Low”). However, since the variant protein lacks higher-order assemblies, the majority of the phase separation–competent protein population was lost. We therefore conclude that G134R-gephyrin exhibited a strongly reduced propensity for LLPS, due to its inability to form higher oligomers.

Importantly, these findings suggest that although receptor binding to the E-domain is preserved, the structural instability of gephyrin assemblies lacking higher oligomers may compromise the integrity of the inhibitory postsynaptic density assuming that G134R-gephyrin fails to generate the protein-dense, phase-separated scaffold required for stabilizing receptor clusters, thereby weakening inhibitory inputs. To directly test this hypothesis in a physiological setting, we next transitioned from in vitro assays to cellular models, first expressing G134R-gephyrin in human embryonic kidney cells (HEK293T; HEK) lacking endogenous gephyrin expression to assess both its oligomeric state, Moco forming activity and whether its altered LLPS propensity manifests as discrete fluorescent condensates in living cells. Building on these experiments, we then extended our analysis to primary hippocampal neurons to determine whether G134R-gephyrin can still support the formation of functional inhibitory synapses.

### In HEK cells, G134R-gephyrin fails to oligomerize, resulting in disrupted Moco synthesis and absence of LLPS

**Figure 5:**
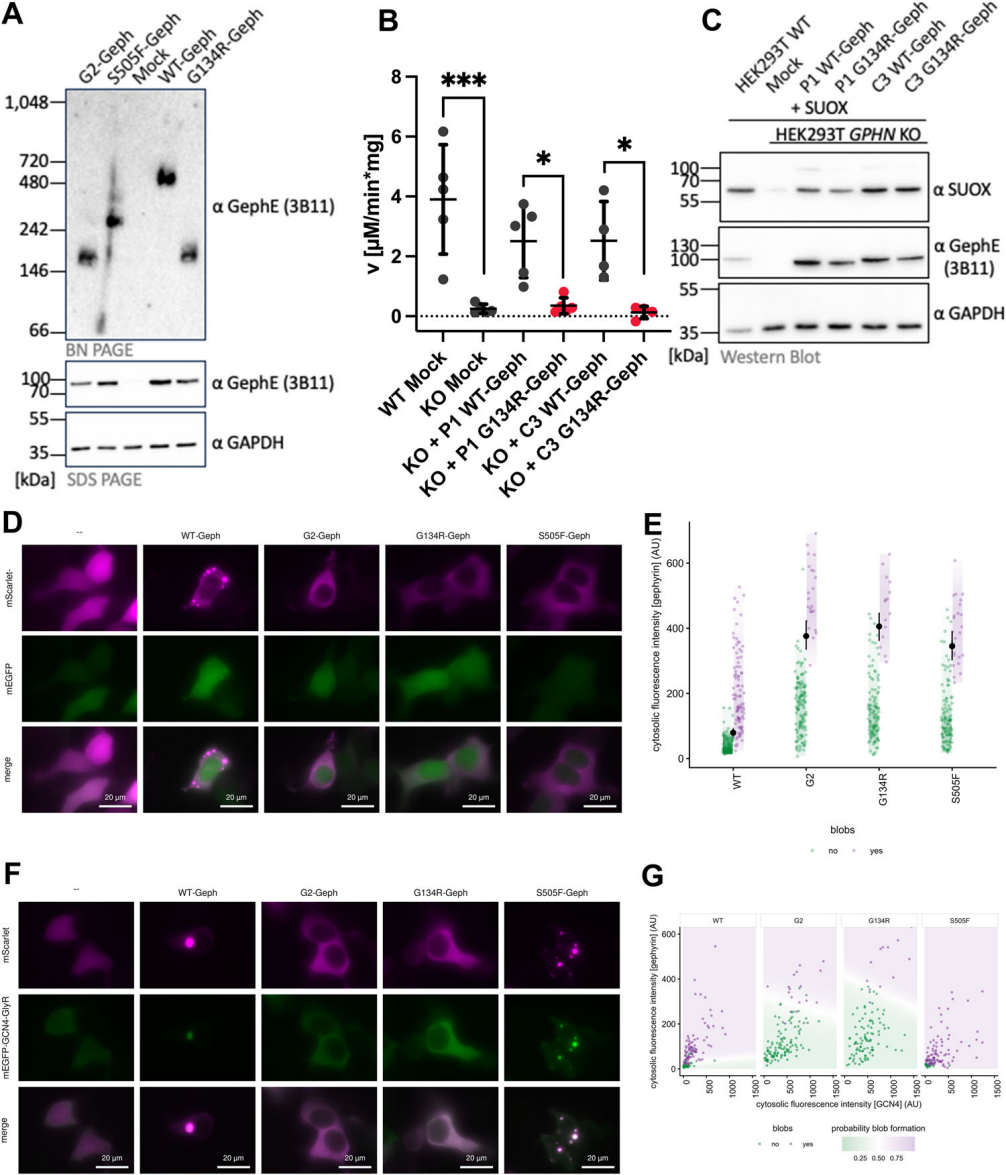
Loss of oligomerization in G134R-gephyrin reduces Moco synthesis and LLPS in HEK cells. (**A**) Representative Western blot analysis after blue native PAGE of HEK *GPHN* KO cells transfected with indicated gephyrin variants. As control Western blot analysis of SDS-PAGE was performed. (**B**) Sulfite oxidase-specific activity in whole cell lysates. Either HEK293T WT (WT) or *GPHN* KO (KO) were transfected with indicated gephyrin variants or empty plasmid (Mock) (*n* = 5 independent experiments; mean ± SD are indicated). Data were analyzed by one-way ANOVA (F_5,22_  =  10.524, *p*  =  2.94 * 10^−5^), Tukey’s post hoc test; p_WT Mock vs. KO Mock_ = 0.000241; p_KO + P1 WT-Geph vs. KO + P1 G134R-Geph_ = 0.0416; p_KO + C3 WT-Geph/KO vs. C3 G134R-Geph_ = 0.0435 (**C**) Representative Western blot analysis of cell lysates from (**B**). (**D**) Representative widefield microscopy images of HEK *GPHN* KO cells expressing mScarlet, mScarlet tagged WT-gephyrin (isoform P1), G2-gephyrin, G134R-gephyrin or S505F gephyrin in the presence of mEGFP. Scale bars represent 20 μm. (**E**) Cytosolic fluorescence intensity of individual mScarlet-gephyrin/mEGFP expressing cells. Data points are color-coded for the presence or absence of cytosolic blobs. The shading density represents probability density, with higher color saturation indicating higher density. Black dots indicate the model-derived point estimate of the onset threshold, defined as the gephyrin intensity corresponding to a predicted 50% probability of blob formation (logistic regression). Error bars display the associated 95% confidence interval. WT 152 cells, G2 144 cells, G134R 127 cells, S505F 129 cells from three independent transfections. (**F**) Representative widefield microscopy images of HEK *GPHN* KO cells expressing mScarlet, mScarlet tagged WT-gephyrin (isoform P1), G2-gephyrin, G134R-gephyrin or S505F gephyrin in the presence of mEGFP tagged GCN4-GlyR. Scale bars represent 20 μm. (**G**) Cytosolic fluorescence intensity of individual mScarlet-gephyrin/mEGFP-GCN4-GlyR expressing cells. Data points are color-coded for the presence or absence of cytosolic blobs. Probability background indicates the combinations of GCN4-GlyR and gephyrin intensities at which the predicted probability of blob formation reaches 50%. WT 131 cells, G2 130 cells, G134R 109 cells, S505F 114 cells from three independent transfections. Source data are available online for this figure.

Our HEK cell-based experiments reveal a severe defect in oligomerization of the G134R-gephyrin variant compared to both WT-gephyrin and previously characterized control variants. Using blue native PAGE, we demonstrate that G134R fails to form native trimers or higher oligomers, running at the same molecular weight as the dimeric control (G2-gephyrin splice variant; (Saiyed et al, 2007; Bedet et al, 2006)), while WT-gephyrin migrates above the trimeric control (S505F-gephyrin E-domain mutant; (Burdina et al, 2025)), confirming its ability to assemble into larger complexes (Fig. 5A). This defect is more pronounced in cells than in vitro, where trimers were still observed. To assess functional consequences, we co-expressed WT- or G134R-gephyrin with sulfite oxidase (SUOX) in HEK gephyrin knockout cells (KO). SUOX activity, which depends on Moco biosynthesis by gephyrin, was restored only by WT-gephyrin, indicating that G134R lacks sufficient catalytic activity for Moco synthesis in this context (Fig. 5B). Similar protein expression was validated via Western blot analysis (Fig. 5C).

Gephyrin forms discrete cytoplasmic condensates in a concentration-dependent manner in cultured mammalian cells, consistent with LLPS (Lee et al, 2024). Similarly, widefield fluorescence microscopy of living HEK gephyrin KO cells revealed that overexpressed mScarlet-tagged WT-gephyrin forms cytosolic blobs (Fig. 5D). Blob formation emerged over a defined range of cytoplasmic fluorescence intensities, consistent with a threshold-like behavior. Using logistic regression, we estimated this onset as the intensity at which the probability of blob formation reached 50%. The estimated onset occurred at 79.6 arbitrary units (AU) of cytosolic gephyrin fluorescence intensity (95% confidence interval (CI): 67.5–93.5 AU) (Fig. 5E). Disrupting G-domain trimerization (G2-gephyrin) and E-domain dimerization (S505F-gephyrin) (Saiyed et al, 2007; Burdina et al, 2025) shifted this onset to higher intensities, with thresholds of 376 AU (95% CI: 334–424 AU) and 345 AU (95% CI: 301–391 AU), respectively (Fig. 5E). Similarly, G134R-gephyrin also exhibited an elevated onset threshold of 406 AU (95% CI: 361–448 AU) (Fig. 5E). Co-expression with inhibitory postsynaptic receptor models lowered the saturation concentration of gephyrin (Lee et al, 2024). Consistent with this, co-expression of the mEGFP-tagged dimeric receptor model GCN4-GlyR promoted mScarlet-tagged WT-gephyrin condensation (Fig. 5F,G). Blob formation depended on the combined abundance of both proteins, as captured by a joint logistic model. To account for this, onset thresholds for gephyrin were estimated at the median GCN4-GlyR intensity within each condition. At median GCN4-GlyR levels, the onset of blob formation with respect to WT-gephyrin intensity occurred at 29.1 AU (95% CI: 22.1–36.0 AU). In contrast, impaired G-domain trimerization markedly increased the onset threshold with values of 301 AU (95% CI: 258–357 AU) for G2-gephyrin and 369 AU (95% CI: 322–435 AU) for G134R-gephyrin (Fig. 5G). By comparison, impaired E-domain dimerization (S505F-gephyrin) showed an onset comparable to WT 15.3 AU (95% CI: 0.507–19.8 AU) (Fig. 5G). Together, these results support the conclusion that G-domain trimerization is critical for gephyrin condensation, whereas E-domain dimerization may even be dispensable in the presence of receptor scaffolding.

These findings indicate that the G134R variant disrupts both oligomerization and phase separation in cells comparative to G2-gephyrin, impairing gephyrin’s metabolic and scaffolding functions. Building on these results, we next aimed to extend our analysis to neuronal cell culture to investigate whether G134R-gephyrin can still support the formation of functional inhibitory synaptic clusters.

### G134R-gephyrin disrupts neuronal clustering and acts dominant negatively when co-expressed with WT-gephyrin

**Figure 6:**
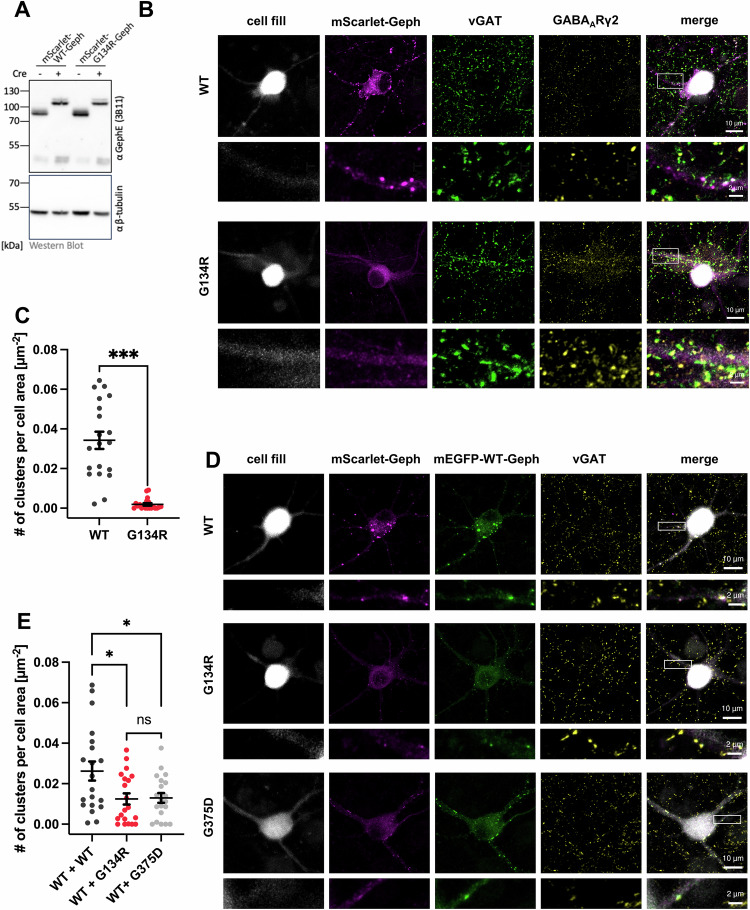
Differential expression of WT- and G134R-gephyrin in primary hippocampal neuron culture. (**A**) Representative Western blot analysis of lysates from gephyrin^floxed/floxed^ murine hippocampal neurons infected with moxBFP (-) or moxBFP-P2A-Cre (+) together with either mScarlet-WT-Geph or mScarlet-G134R-Geph. (**B**) Representative confocal images of gephyrin^floxed/floxed^ murine hippocampal neurons transfected with moxBFP-P2A-Cre and infected with either mScarlet-WT-Geph or mScarlet-G134R-Geph and immunostained against vesicular GABA transporter (vGAT, green) and GABA_A_R γ2 subunit (yellow). (**C**) Quantification of mScarlet-tagged gephyrin clusters was carried out using automated synapse analyses. Each data point represents synaptic cluster density (# per cell area µm^−2^) per acquired cell; *n*  =  20 cells per condition from five independent cultures, mean ± SD are indicated. Data were analyzed by Mann–Whitney U test U = 391, *p* = 0.00000023 (***), effect size r = 0.820 (**D**) Representative confocal images of gephyrin^floxed/floxed^ murine hippocampal neurons transfected with moxBFP-P2A-Cre and infected with mEGFP-WT-Geph and either mScarlet-WT-Geph or mScarlet-G134R-Geph and immunostained against vesicular GABA transporter (vGAT, yellow). (**E**) Quantification of mScarlet-tagged gephyrin clusters was carried out using automated synapse analyses. Each data point represents synaptic cluster density (# per cell area µm^−2^) per acquired cell; *n*  =  20 cells per condition from five independent cultures, mean ± SD are indicated. Data were analyzed by one-way ANOVA (F_2,57_  =  5.139, *p*  =  0.009), Tukey’s post hoc test; p_WT + WT vs. WT + G134R_ = 0.0176 (*); p_WT + WT vs. WT + G375D_ = 0.0228 (*); p_WT + G134R vs. WT + G375D_ = 0.995 (ns). Source data are available online for this figure.

We finally tested the G134R variant in primary hippocampal neurons. To achieve expression in cells lacking endogenous gephyrin, we generated dissociated hippocampal cultures from floxed gephyrin mice (O’Sullivan et al, 2016). Knockout of endogenous gephyrin was achieved by Cre expression, which was combined with Cre-dependent expression of mScarlet-tagged WT- or G134R-gephyrin (Fig. 6A). Under these conditions, WT-gephyrin formed robust somatic and dendritic clusters colocalizing with the presynaptic inhibitory marker vGAT and the γ2 subunit of GABA_A_R, confirming their synaptic identity (Fig. 6B,C). In contrast, neurons expressing only G134R-gephyrin displayed a dramatic loss in the number of synaptic clusters, with markedly fewer puncta colocalizing with vGAT and γ2 (Fig. 6B,C). These results demonstrate that G134R-gephyrin is unable to fully support the assembly of inhibitory postsynaptic scaffolds in neurons by itself.

To model the heterozygous state found in the patient, we tested whether the G134R variant exerts a dominant-negative effect on WT-gephyrin, potentially contributing to the observed epileptic phenotype. We co-expressed mEGFP-tagged WT-gephyrin and mScarlet-tagged G134R in neurons, enabling clear distinction between the two proteins based on their respective fluorescent labels. As a positive control, we included the previously described dominant-negative variant G375D (Fig. 6D). Heterozygous expression of G134R significantly reduced the density of WT inhibitory postsynaptic clusters to a similar extent as G375D, supporting a dominant-negative effect (Fig. 6E). These findings indicate that G134R-gephyrin interferes with WT-gephyrin function, providing a plausible molecular mechanism for the patient’s epileptic phenotype.

## Discussion

Our study identifies G134R-gephyrin as the first epilepsy patient-derived variant from a missense mutation in the *GPHN* gene affecting the G-domain of gephyrin, demonstrating that a single amino acid substitution disrupts gephyrin’s oligomerization and LLPS. However, we acknowledge the inherent limitations of panel-based next-generation sequencing (NGS), as variants outside the captured regions cannot be formally excluded. Previously, two missense mutations affecting gephyrin’s G-domain, namely V43L and A91T were identified in patients with autism spectrum disorder (Lionel et al, 2013). However, functional analyses revealed only moderate clustering deficiencies for A91T, while V43L behaved normally under the experimental conditions (Kim et al, 2021). Recombinant and purified G134R-gephyrin formed trimeric lower-order oligomers, shows reduced Moco synthesis, while a dominant-negative effect on its enzymatic activity was not observed as expected from the patient’s biomarker profile. While we could not resolve the structural consequences of this variant at atomic resolution, our aim was to provide an integrative functional characterization that advances the understanding of this disease-associated mutation, and could even go beyond by identifying key and novel principles of gephyrin-dependent phase separation at the postsynaptic density (PSD). However, in a human cellular model, G134R-gephyrin failed to form higher-order oligomers beyond E-domain mediated dimerization and lost its Moco synthesis function entirely. While G134R-gephyrin retains the ability to interact with the intracellular domain of the glycine receptor, it is unable to form synaptic clusters in dissociated hippocampal neurons, both in a homozygous and heterozygous context, where it acts dominantly negative on WT-gephyrin; although these analyses are limited to neuronal cell culture and an entire knock-in mouse model would provide a more comprehensive in vivo assessment, they nevertheless allow us to evaluate synaptic targeting and clustering in a native neuronal context. This dominant-negative effect, which mirrors the behavior of the epileptogenic E-domain mutant G375D (Dejanovic et al, 2015), provides a direct molecular explanation for the patient’s epileptic phenotype by effectively depleting functional inhibitory receptor-scaffold complexes at synapses.

**Figure 7:**
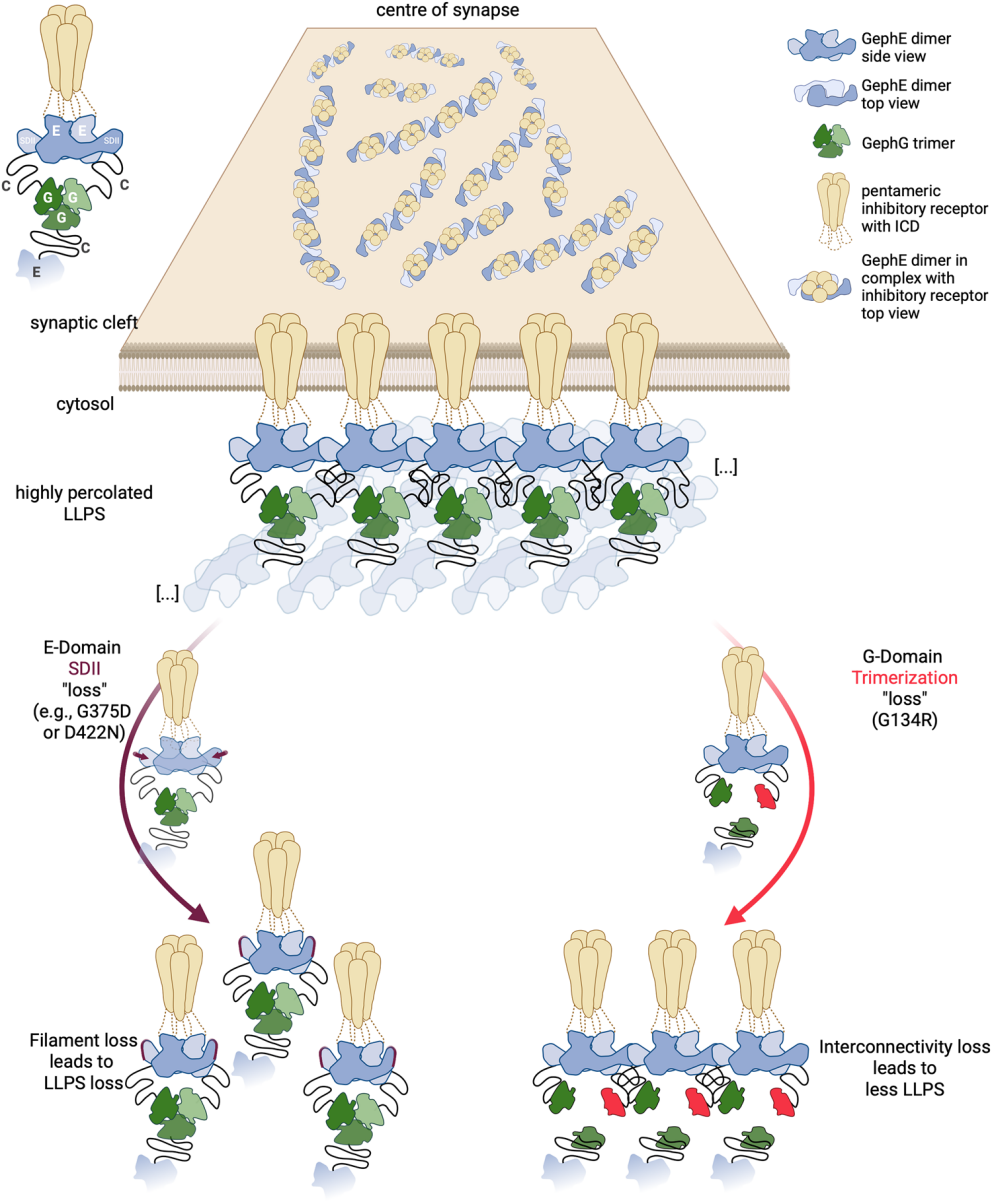
Refined model for gephyrin LLPS. Schematic illustrating the proposed impact of disease-associated gephyrin variants on higher-order network assembly and LLPS. (**Centre**) WT gephyrin forms a highly percolated network through coordinated G-domain trimerization and E-domain dimerization, which we hypothesize is required for efficient receptor immobilization and stable postsynaptic clustering. (**Left**) Previously characterized E-domain variants affecting subdomain II (G375D and D422N) disrupt filament formation and impair LLPS as reported in the literature. (**Right**) The newly identified G134R variant, located in the G-domain, is proposed to selectively disrupt G-domain-mediated intermolecular connectivity without affecting E-domain interactions, thereby reducing network percolation and impairing higher-order assembly and LLPS. Together, this model suggests that both G- and E-domain interactions contribute to the percolation threshold required for gephyrin condensate formation and functional synaptic clustering (Figure created with BioRender).

Previous work from our laboratory identified the two E-domain gephyrin variants G375D and D422N in patients with developmental epilepsy. Both variants perturb LLPS, thus establishing the E-domain as a crucial structural hub for condensate formation and inhibitory synapse integrity (Dejanovic et al, 2015; Macha et al, 2022, 2025). Structural and functional analyses revealed that the subdomain two (SDII) within the E-domain is essential for the recently discovered filament formation via SDII–SDII’ interactions, which in turn underlie LLPS and define the structural basis of postsynaptic gephyrin scaffolds (Macha et al, 2025). The G375D variant, located within SDII, causes unfolding of this subdomain, abolishing SDII–SDII’ mediated filament formation and thereby eliminating LLPS and synaptic clustering capacity (Macha et al, 2025). In contrast, the D422N variant disrupts a critical salt bridge with R379, yet preserves the overall SDII fold, still resulting in loss of LLPS and impaired inhibitory synaptic transmission, highlighting that subtle perturbations of the SDII network suffice to destabilize condensate formation (Fig. 7 left).

Taken together with our current observations, we propose a refined model for establishing the postsynaptic architecture: while the E-domain is the primary driver of phase separation and provides the receptor-binding interface, the G-domain is crucial for interconnecting E-domain gephyrin conjugates into higher-order oligomers, thereby amplifying LLPS capacity and stabilizing synaptic clustering (Fig. 7 right). The strong G-domain trimerization (Herweg and Schwarz, 2012) may provide a hierarchical organizing principle that ensures E-domain dimers efficiently find and engage each other, thereby reaching the threshold concentrations required for LLPS and filament maturation under physiological conditions. Simultaneously, E-domain dimers nucleate filaments through SDII–SDII’ interactions, and G-domain trimerization could interlink these E-domain-based filaments into a robust three-dimensional scaffold that can maintain the protein concentrations and geometric connectivity observed in vitro by cryo-EM and required in vivo at synapses. Within this framework, G134R-gephyrin is as functionally disruptive at the cellular and clinical levels as the E-domain SDII variant G375D, despite residing outside the critical receptor binding and filament-forming domains of gephyrin, indicating that G-domain-mediated higher-order oligomerization is equally important as SDII-dependent filament assembly for LLPS and synapse formation.

Our convergent mechanistic insight suggests an additional evolutionary advantage for the fusion of G- and E-domains via the flexible C-domain in vertebrates within a single polypeptide chain: it serves both, product-substrate-channeling in Moco synthesis (Belaidi and Schwarz, 2013) and precise control over condensate assembly for postsynaptic receptor clustering. The flexible C-domain likely permits the G-domain trimeric core to be spatially separated from the E-domain filament lattice, enabling dynamic regulation of scaffold connectivity without steric interference with filament formation.

The central C-domain of gephyrin, which behaves as an intrinsically disordered region (IDR), is indispensable for scaffold condensation and can be functionally replaced by heterologous IDRs, in some cases even enhancing gephyrin’s LLPS capacity, indicating that the C-domain acts as a charged spacer that thermodynamically modulates phase separation rather than as a classical self-associating IDR (Lee et al, 2024). In apparent contrast, segments of the C-domain can inhibit LLPS driven by the E-domain in vitro, suggesting that under these conditions C-domain–E-domain contacts can restrain E-domain phase separation and thus provide a built-in brake on spontaneous condensation (Bai et al, 2021). Integrating these findings, the conformation and exposure of the C-domain operates as a dynamic hub that senses local receptor density and postsynaptic architecture, defining the boundary of the inhibitory postsynaptic density so that gephyrin condensates remain confined to receptor-enriched nanodomains, while remaining permissive to rapid remodeling. In this scenario, the G-domain not only interconnects E-domain filaments but also allosterically controls C-domain compaction *versus* extension, thereby toggling between C-domain–shielded states that limit LLPS and C-domain–exposed states in which favorable electrostatic C–E interactions and increased effective valency promote percolation of a gephyrin network. This polydisperse nature of gephyrin conformation has been seen already in early structural studies using small-angle X-ray scattering (Sander et al, 2013). From the perspective of percolation-based phase separation hypothesis (Mittag and Pappu, 2022; Shen et al, 2023), where the condensed phase emerges once a system-spanning, multivalent interaction network forms, the G-domain-mediated trimerization becomes a fundamental requirement: by providing strong, high-valency cross-links between SDII-based E-domain filaments, G-domain connectivity helps to drive the system across the percolation threshold into a macroscopically connected, viscoelastic condensate, as described for other synaptic and PSD-like assemblies (Zhu et al, 2024) (Fig. 7). Consequently, pathogenic disruption of G-domain trimerization, as here discovered for G134R-gephyrin, is predicted not only to lower LLPS propensity but to prevent formation of a percolated scaffold network that is necessary to immobilize and concentrate inhibitory receptors within the postsynaptic density, thereby unifying G-domain and E-domain mutations within a common percolation framework for gephyrin-dependent inhibitory synapse failure.

The observation that both E-domain (G375D, D422N) and G-domain (G134R) epilepsy variants converge on a shared deficit in gephyrin LLPS implies that fine-tuning of G-domain trimerization and E-domain filament assembly constitutes a key regulatory axis to strengthen or weaken inhibitory synaptic inputs. Such multilayered regulation of filament formation, trimer crosslinking, and LLPS likely equips gephyrin with the ideal biophysical properties to function as a tunable postsynaptic scaffold in neurons, while at the same time making the system exquisitely sensitive to disease-causing missense perturbations in both the G- and E-domains.

## Methods

**Table:** Reagents and tools table

Reagent/Resource	Reference or Source	Identifier or Catalog Number
**Experimental models**		
floxed gephyrin (*M. musculus*)	O’Sullivan et al, 2016	
C57BL/6NRj (*M. musculus*)	Janvier Labs	
HEK293T	DSMZ	ACC 635
HEK293T *GPHN* knockout	Burdina et al, 2025	
*E. coli* BL21 (DE3)	Invitrogen	C600003
**Recombinant DNA**		
pAAV-hSyn-FLEX-mScarlet-Gphn_P1	Macha et al, 2025	
pAAV-hSyn-FLEX-mScarlet-Gphn_P1_G375D	Macha et al, 2025	
pAAV-CamKIIa-moxBFP-P2A-Cre	Macha et al, 2025	
pAAV-hSyn-FLEX-mScarlet-Gphn_P1_G134R	This study	
pAAV-hSyn-FLEX-mEGFP-Gphn_P1	This study	
pAdDeltaF6	Addgene	# 112867
pAAV2/1	Addgene	# 112862
pcDNA™3.1/Zeo (+)	Invitrogen	V86520
pcDNA™3.1/Zeo (+) Gphn_P1	This study	
pcDNA™3.1/Zeo (+) Gphn_G2	This study	
pcDNA™3.1/Zeo (+) Gphn_S505F	This study	
pcDNA™3.1/Zeo (+) Gphn_G134R	This study	
pcDNA™3.1/Zeo (+) Gphn_C3	This study	
pcDNA™3.1/Zeo (+) Gphn_C3_G134R	This study	
human sulfite oxidase in pcDNA5 FRT	Bender et al, 2019	
pmEGFP-C2	Liebsch et al, 2023	
pmEGFP-C2-GCN4-GlyR	This study	
pmScarlet-C2	This study	
pmScarlet-C2 Gphn_P1	This study	
pmScarlet-C2 Gphn_G2	This study	
pmScarlet-C2 Gphn_S505F	This study	
pmScarlet-C2 Gphn_G134R	This study	
pET-32M3C His6-GCN4-GlyR-βLD	Bai et al, 2021	
pET-32M3C His6-GCN4-GABAAR-α1LD	Bai et al, 2021	
pET-32M3C His6-GCN4-GABAAR-α3LD	Bai et al, 2021	
pQE80 Gephyrin P1	This study	
pQE80 Gephyrin P1 G134R	This study	
pQE80 Gephyrin G-domain	This study	
pQE80 Gephyrin G-domain G134R	This study	
**Antibodies**		
Anti-vesicular GABA transporter (vGAT)	Synaptic Systems	#131003
Anti-GABA_A_R γ2	Synaptic Systems	#224004
Goat anti-rabbit AlexaFluor 488	Thermo Fisher Scientific	#A-11034
Goat anti-rabbit AlexaFluor 647	Thermo Fisher Scientific	#A-21245
Goat anti-guinea pig AlexaFluor 647	Abcam	#ab150187
Anti-GephE (3B11)	Self-made	RRID: AB_887719
Anti-SUOX	Sigma	WH0006821M1
Anti-GAPDH	Sigma	G9545
Anti-Tubulin	Cell Signaling	#2128
Anti-mouse HRP-coupled	Sigma	AP181P
Anti-rabbit HRP-coupled	Sigma	AP187P
**Oligonucleotides and other sequence-based reagents**		
GACCAGTGTGTAGAATAAGAGGGAAAA	G134R fw	
TTTTCCCTCTTATTCTACACACTGGTC	G134R rev	
CTCATTCCCTGTGAACATAACGGCAAC	S505F fw	
GTTGCCGTTATGTTCACAGGGAATGAG	S505F rev	
CACGAGATGTCACTCCAGAGAAATTCCCAACATTCCCATTTTGTGGGC	G2 fw	
CCCGTTCTATTACTTCTTTTGTGGCCCCTTTCTGGAGCCCACAAAATGGGAAT	G2 rev	
GACCGAGGGAATGATCCT	Geph flanking fw	
AGGATCATTCCCTCGGTC	Geph flanking rev	
GACTCAGATCTCGAGCATGGCGACCGAGGGAAT	pAAV G134R subcloning fw	
TAGCCGTCCGATGACC	pAAV G134R subcloning rev	
**Chemicals, Enzymes and other reagents**		
Gel green®	Biotium	#41005
MgCl_2_	VWR Chemicals	25107.292
Benzonase	EMD Millipore	70664-10KUN
Lipofectamine 2000	Thermo Fisher Scientific	11668500
Neurobasal medium	Thermo Fisher Scientific	21103049
B-27	Thermo Fisher Scientific	17504044
N-2	Thermo Fisher Scientific	17502048
GlutaMAX^TM^	Thermo Fisher Scientific	35050061
PEI	Sigma	408727
Poly-L-lysine	Sigma	P1399
Pen/Strep	Pan Biotech	P06-07100
PEG 8000	Sigma	P2139
Na_2_HPO_4_	Sigma	S5136
KH_2_PO_4_	Roth	3904.3
Paraformaldehyde	AppliChem	A3813
Goat serum	Pan Biotech	P30-1002
Triton-X100	Roth	3051.2
Mowiol	Calbiochem	475904
Dabco	Merck	8.03456.0100
BSA	Biomol	01400.100
NH_4_Cl	Roth	K298.2
Poly-D-lysine	Sigma	P6407
cOmplete protease inhibitor	Roche	4693116001
Bicinchoninic Acid	Sigma	BCA1-1KT
Superdex 16/600 prep grade	GE Healthcare	GE28-9893-35
4%–16% native PAGE	TM Novex, Invitrogen	BN1002BOX
Catalase	Sigma-Aldrich	219261
Ampicillin	Roth	K029.2
Tryptone	Pronadisa	1612
Yeast extract	Pronadisa	1702
NaCl	AppliChem	A4256
KCl	Sigma	P5405
IPTG	Inalco	1758-1400
Tris	Sigma	T1503
HCl	Prolabo	20255.290
Tween20	Applichem	A1389
β-mercaptoethanol	Sigma	M6250
DNAse	Roche	10104159001
Lysozyme	Sigma	62971-10G-F
Ni-NTA	Cube Biotech	31103
Imidazole	Roth	3899.4
Glycerol	Roth	70530.4
SDS	AppliChem	A7249
Acrylamide 30%	Roth	3029.1
Bromophenol blue	Merck	8122
Ethanol	Prolabo	20821.330
Acetic acid	Merck	8.18755.2500
Coomassie brilliant blue R250	VWR	0472
SuperSignal™ West Pico PLUS Chemilumineszenz-Substrat	Thermo Fisher Scientific	34580
6-aminohexanoic acid	Sigma	A2504
Coomassie brilliant blue G250	VWR	443293X
EDTA	Roth	CN06.3
Dulbecco’s modified Eagle’s medium	Pan Biotech	P04-03500
FBS	Pan Biotech	P40-37500
Glutamine	Pan Biotech	P04-80050
HEPES	Roth	HN78.3
DDM	GlyconBioch	D97002-C
IGEPAL	Sigma	18896
Deoxycholate	Sigma	D6750
Cytochrome *c*	Biosynth	FC32167
KCN	Merck	1.04967.01.00
Na_2_SO_3_	Sigma	S0505
**Software**		
BioRender	https://app.biorender.com	
ChimeraX-1.8	Version -1.8	
Perplexity	https://www.perplexity.ai	
RStudio	R version 4.5.2	
GraphPad Prism 10	Version 10.2.3	
Python	Version 3.10.8	
AlphaFold3	https://alphafoldserver.com/welcome Jumper et al, 2021	
BioRad Image Lab	Version 6.1	
OriginLab Corporation	Origin 7	
**Other**		

### Human subject

Parents/legal guardians of subject included in this study signed an informed consent form for participation. All experiments conformed to the principles set out in the WMA Declaration of Helsinki and the Department of Health and Human Services Belmont Report.

### Expression constructs

6His-tagged gephyrin and GlyR β-loop-intein expression vectors have been described before (Macha et al, 2022; Dejanovic et al, 2015). GCN4-based GlyR-ICD and GABA_A_R-ɑ1/3-ICD were kindly provided by the Zhang lab (Bai et al, 2021). 6His-tagged gephyrin G-domain and full-length gephyrin (isoforms P1 and C3 in pQE80) G134R variants were cloned by site-directed mutagenesis using forward (GACCAGTGTGTAGAATAAGAGGGAAAA) and reverse (TTTTCCCTCTTATTCTACACACTGGTC) primers. 6His-tagged full-length gephyrin (isoforms P1 in pQE80) S505F variant was cloned by site-directed mutagenesis using forward (CTCATTCCCTGTGAACATAACGGCAAC) and reverse (GTTGCCGTTATGTTCACAGGGAATGAG) primers. 6His-tagged full-length gephyrin G2 in pQE80 was cloned by overlap extension PCR using G2 forward (CACGAGATGTCACTCCAGAGAAATTCCCAACATTCCCATTTTGTGGGC) and G2 reverse (CCCGTTCTATTACTTCTTTTGTGGCCCCTTTCTGGAGCCCACAAAATGGGAAT) with gephyrin flanking forward (GACCGAGGGAATGATCCT) and flanking reverse (AGGATCATTCCCTCGGTC) primers. Full-length gephyrin and its variants were subcloned into pcDNA™3.1/Zeo (+) (Invitrogen) using NEBuilder HiFi DNA Assembly (NEB). Gephyrin was initially subcloned into pmEGFP-C2 (Liebsch et al, 2023), derived from pEGFP-C2 (Clonetech) using XhoI and KpnI restriction sites. mScarlet-Gphn constructs in this plamid were generated by replacing EGFP with mScarlet from pAAV-hSyn-mScarlet (a gift from Karl Deisseroth, Addgene plasmid #131001; http://n2t.net/addgene:131001; RRID:Addgene_131001) using NEBuilder HiFi DNA Assembly (NEB). mEGFP-GCN4-GlyR in pEGFP-C2 (Clonetech) was generated by PCR amplification of GCN4-GlyR and subcloned into pmEGFP-C2 using XhoI and KpnI. Full-length gephyrin (isoform P1) G134R was amplified with forward (GACTCAGATCTCGAGCATGGCGACCGAGGGAAT) and reverse (TAGCCGTCCGATGACC) primers and subcloned into pAAV-hSyn-FLEX-mScarlet-Gphn_P1 (Macha et al, 2025) using XhoI and BshTI restriction sites. mEGFP-tagged full-length gephyrin (isoforms P1) was subcloned into pAAV-hSyn-FLEX-mScarlet-Gphn_P1 using NcoI restriction site. The plasmids pAdDeltaF6 (Addgene plasmid # 112867; RRID:Addgene_112867) and pAAV2/1 (Addgene plasmid # 112862; RRID:Addgene_112862) were gifts from James M. Wilson. All DNA constructs were confirmed by Sanger sequencing (Eurofins), and the integrity of AAV2 ITR sites was confirmed by analytical SmaI restriction digest.

### Protein expression, purification and size exclusion chromatography (SEC)

All gephyrin variants used in this study were recombinantly expressed in *E. coli* BL21 (DE3) rosetta cells in 1x LB medium containing 0.1 mg/ml ampicillin. Prior to expression, cells were grown until OD600 of 0.3–0.4 was reached. His-tagged full-length gephyrin was expressed for 16 h at 18 °C. His-tagged GephG was expressed for 16 h at 30 °C. Protein expression was induced by addition of isopropyl-β-D-thiogalactopyranosid (IPTG) to an end-concentration 250 μM in the cell-culture. After protein expression, cells were harvested by centrifugation at 4000 × *g* and resuspended in lysis buffer (100 mM Tris/HCl pH 8, 250 mM NaCl, 0.05% (v/v) Tween20, 5 mM β-mercaptoethanol, 1x protease inhibitor (cOmplete, Roche, Basel, Switzerland), 6.5 mg lysozyme per L expression culture from chicken egg (Sigma-Aldrich) and 25 µg DNAse per mL lysis Buffer) and stored at −20 °C for further use. For purification, the cells were lysed mechanically using EmulsiFlex (Avestin, Ottawa, Canada) and ultrasound for 3× 30 s at 45% amplitude. Cytosolic extract was separated from cell debris by centrifugation at 50,000 × *g* for 45 min. Prior to Ni-NTA chromatography, the cell extracts were supplemented with imidazole to 20 mM end concentration. The cell extract was applied to 10 ml Ni-NTA resin and proteins were washed by 10 column volumes (CV) of lysis buffer, 10 CV of lysis buffer containing 10 mM imidazole and 10 CV of lysis buffer containing 20 mM imidazole. Proteins were eluted with 3 CV lysis buffer containing 400 mM imidazole. All proteins were further purified and analyzed via size exclusion chromatography (SEC) (Superdex 16/600 prep grade, GE Healthcare) using protein buffer (20 mM Tris/HCl pH 7.5, 250 mM NaCl, 5 mM β-mercaptoethanol, 5% glycerol) at a flow rate of 1 mL/min. All purification steps were performed at 4 °C. GlyR-βloop was expressed and purified as stated previously (Grünewald et al, 2018). GlyR-βICD, GABA_A_R-α1ICD, and GABA_A_R-α3ICD were purified according to the protocol provided by Bai and colleagues (Bai et al, 2021).

### Isothermal titration calorimetry (ITC)

All experiments were performed with a MicroCal Auto-ITC200 (Malvern, Malvern, United Kingdom) in ITC buffer (20 mM Tris/HCl pH 7.5, 250 mM NaCl, 5 mM β-mercaptoethanol, 5% glycerol). The sample cell was filled with gephyrin variants at concentration of 20–30 μM. GlyR-βloop was filled into the syringe at a concentration of 250–350 μM. Experiments were conducted at 37 °C with an injection volume of 1.5–2 μl, a set reference power of 5 μcal/s, spacing time of 180–300 s and a stirring speed of 750 rpm. Data was analyzed using Origin 7 (OriginLab Corporation) and binding parameters were derived by applying a two-site binding model.

### Sedimentation assay of in vitro phase separation

Prior the assay proteins were pre-cleared at 17,000× *g* for 10 min at 25 °C. For sedimentation assays, proteins were mixed at designated combinations and with 10 µM gephyrin and 20 µM receptor model in 50 μL final volume in assay buffer (25 mM Tris pH 7.4, 150 mM NaCl). After 10 min incubation at 25 °C, the mixtures were centrifuged at 17,000× *g* for 10 min at 4 °C. Right after centrifugation, the supernatant and pellet were separated. The pellet was brought to the same volume as the supernatant. Proteins recovered in supernatant and pellet were analyzed by SDS-PAGE with Coomassie Blue staining. The band intensities in SDS-PAGE gels were quantified by Image Lab software. Relative and absolute amounts of proteins in supernatant and pellet were calculated based on the relative band intensities. Data of three repeats were presented as means ± SD.

### In vitro Moco synthesis assay

The in vitro Moco synthesis assay has previously been described in detail (Belaidi and Schwarz, 2013). About 150 pmol *E. coli*-expressed and purified gephyrin was used per condition. Samples without gephyrin or without molybdenum were used as control and show Moco produced either chemically or by trace amounts of molybdenum in the buffers. Proteins from two independent purifications were used for the assay.

### SDS-PAGE, Coomassie, and Western Blot

Samples were supplemented with 1× sample buffer (5×: 250 mM Tris/HCl pH 6.8, 30% glycerol, 0.1% Bromophenol blue, 10% SDS, 5% β-mercaptoethanol) and incubated for 5 min at 95 °C. Protein separation was performed with 12% SDS acrylamide gels, followed by Coomassie staining (30% EtOH, 10% acetic acid, 0.25% Coomassie brilliant blue R250) or immunoblotting using standard protocols with a chemiluminescence system. The following antibodies were used and diluted in Tris-buffered saline/0.5% Tween containing 1% dry milk: anti-gephyrin E-domain (3B11, 1:20, self-made, RRID: AB_887719); anti-GAPDH (1:1000, G9545, Sigma), anti-mouse HRP-coupled (1:10,000, AP181P, Sigma); anti-rabbit HRP-coupled (1:10,000, AP187P, Sigma). Image acquisition of Coomassie stained gels and immunoblot detection were performed with a ChemiDocTM Imaging System (BioRad). Band intensities were quantified using Image Lab 6.1 (BioRad).

### Blue native PAGE analysis

Samples for blue native PAGE analysis were prepared by 1:3 dilution using H_2_O and the addition of 10× blue native loading buffer (312 mM imidazole, 500 mM 6-aminohexanoic acid, 5% CBB G250, 6.25 mM EDTA, 40% glycerol). Samples were applied to 4–16% native PAGE (TM Novex Bis-Tris Gel) and protein separation was performed according to the manufacturer’s protocol. Afterward, gels were subjected to Western blotting.

### Structural predictions by AlphaFold3

Structural predictions were run in AlphaFold3 (Jumper et al, 2021). For structural comparison the GephG WT (PDB ID: 1JLJ) was used. For G134R substitution the gephyrin WT (UniProt ID: Q9NQX3) sequence was used. Predictions were run three times, with all five output predictions used for analysis. Sequence alignments were conducted on the UniProt website (The UniProt Consortium 2025).

### Generation and transfection of HEK293T *GPHN* KO Cells

HEK293T (DSMZ no. ACC 635) and HEK293T *GPHN* KO cell were not recently authenticated but are tested annually for mycoplasma contamination. Generation of HEK293T *GPHN* KO cells was described by N. Burdina and colleagues (Burdina et al, 2025). 1 × 10^6^ HEK293T *GPHN* KO cells were plated per well into 6-well cell culture plates or 2 × 10^5^ HEK293T *GPHN* KO cells were plated on poly-D-lysine (Sigma) coated 35 mm glass-bottom dishes (ibidi) and incubated 24 h in Dulbecco’s modified Eagle’s medium supplemented with 10% FCS and 2 mM l-glutamine at 37 °C and 5% CO_2_. Transfection was performed using 200 ng (per well in 6-well) or 1000 ng (per 35 mm glass-bottom dish) of the respective plasmid DNA with polyethylenimine (PEI, Sigma) according to the manufacturer’s protocol. 14 h after PEI transfection, cells were washed twice in ice-cold PBS and lysed in native lysis buffer (20 mM HEPES pH 8.0, 150 mM NaCl, 5% Glycerol, 5 mM EDTA, 1% DDM, 2x protease inhibitor (cOmplete, Roche, Basel, Switzerland)). Lysates were cleared at 4 °C and 14,000 × g for 20 min and total protein concentration determined using Bicinchoninic Acid assay (Sigma). 16–18 h after PEI transfection live cells were imaged using a Nikon Ti2-E microscope (see below).

### Sulfite oxidase activity in *GPHN* KO HEK293T cells

HEK293T cells and *GPHN* KO HEK293T cells were seeded in 6-well plates and co-transfected the next day with human sulfite oxidase in pcDNA5 FRT (Bender et al, 2019) and untagged gephyrin constructs (or empty plasmid, Mock) in pcDNA™3.1/Zeo (+) using Polyethylenimine. 24 h post-transfection cells were washed with PBS and lysed for 1 h at 4 °C (150 mM NaCl, 5 mM EDTA, 1% (w/v) IGEPAL, 0.5% (w/v) desoxycholate, 0.1% (w/v) SDS, 50 mM Tris/HCl pH 7.5 with 1x protease inhibitor mix (cOmplete, Roche, Basel, Switzerland)). Lysates were cleared at 4 °C and 14,000 × *g* for 20 min and total protein concentration determined using Bicinchoninic Acid assay (Sigma). Sulfite oxidase-specific activity in 100 µg whole cell lysate was measured at room temperature (aerobic conditions) using a plate reader equipped with monochromators (Tecan Spark). In 200 μL, 50 μM cytochrome *c*, 20 μg/mL catalase (bovine, Sigma-Aldrich), 50 µM KCN, and 300 μM Na_2_SO_3_ were combined in 100 mM Tris/Ac pH 8.0 and reduced cytochrome *c* was measured at 550 nm. Initial cytochrome *c* reduction velocities were determined and specific activities calculated using Lambert-Beer Law with ε_550 nm_ = 19 630 M^−1^ cm^−1^ and *d* = 0.857 cm.

### Primary hippocampal cultures

Floxed gephyrin mice (O’Sullivan et al, 2016), a gift from Ralph A. Nawrotzki, were back-crossed to C57BL/6NRj. Mice were housed on a 12 h light-dark cycle with free access to food and water at 22  ±  2 °C and a humidity of 55  ±  10%. Dissociated primary hippocampal cultures from homozygous gephyrin floxed embryos (E17.5) were prepared of either sex. Embryonal hippocampi obtained from one pregnant mouse were pooled prior to cell dissociation. After dissociation, 65,000 cells were seeded on Poly-L-lysine-coated 13-mm cover slips or 91,000 cells per well on Poly-L-lysine-coated 24-well plates. Cultures were grown in 0.5 mL per well neurobasal medium supplemented with B-27, N-2, GlutaMAX^TM^ (Thermo Fisher Scientific), and Pen/Strep. Recombinant AAV1 particles were diluted in Neurobasal medium with supplements before they were added to the cultures. Cultures were infected (for Western blot analysis) with 1 × 10^8^ viral genome copies (GC) of moxBFP or moxBFP-P2A-Cre or transfected (for immunocytochemistry) with 400 ng DNA using Lipofectamine 2000 after 6 days in vitro (DIV) and with 5 × 10^8^ GC of AAV-hSyn-FLEX-mScarlet constructs after 9 DIV. We complied with all relevant ethical regulations for animal testing and research and experiments were approved by local ethics committees (Germany, Landesamt für Natur, Umwelt und Verbraucherschutz Nordrhein-Westfalen, reference 2021.A450). The study is reported in accordance with the ARRIVE guidelines.

### rAAV2/1 preparation

Recombinant AAV2/1 particles were prepared in HEK293T cells (DSMZ no. ACC 635), precipitated with PEG/NaCl, and subsequently cleared via chloroform extraction (Kimura et al, 2019). Purity of preparations was assessed with SDS-PAGE, and titers were determined using Gel green® (Biotium) (Xu et al, 2020).

### Immunocytochemistry

Cells were washed with PBS and fixed at DIV17 with 4% formaldehyde in PBS for 15 min and quenched with NH_4_Cl in PBS at room temperature. After blocking/permeabilization for 1 h with 10% goat serum, 1% BSA, 0.2% Triton X-100 in PBS, the following primary antibodies (Synaptic Systems) were used: anti-vesicular GABA transporter (vGAT) (1:1000, #131003) for inhibitory presynaptic terminals; anti-GABA_A_R γ2 (1:500, #224004) for postsynaptic GABA_A_Rs. The following secondary antibodies were used: goat anti-rabbit AlexaFluor 488 (1:500, #A-11034, Thermo Fisher Scientific), goat anti-rabbit AlexaFluor 647 (1:500, #A-21245, Thermo Fisher Scientific), and goat anti-guinea pig AlexaFluor 647 (1:500, #ab150187, Abcam). Coverslips were mounted with Mowiol/Dabco.

### Widefield microscopy and image analysis

Image stacks [0.3-μm z-step size, 1600 × 1200 (281.90 × 211.43 μm)] were acquired on a Nikon Ti2-E inverse microscope with an Apo 60x Oil λ S DIC N2 objective, equipped with TxRed(Ex560/Em630) and EGFP(Ex480/Em535) filter cubes and a QImaging Retiga 2000R Mono 12-bit CCD camera. A random forest classifier was used for pixel classification (binarization) of blobs. Cytoplasmic fluorescence intensities were manually measured from background corrected average intensity Z-projections. Blob formation (binary outcome) was modeled as a function of cytoplasmic fluorescence intensity using logistic regression. The onset threshold was defined as the intensity corresponding to a predicted 50% probability of blob formation. Logistic regression models including both gephyrin and GCN4-GlyR cytoplasmic fluorescence intensities were used to estimate blob formation probability. The onset threshold for gephyrin was defined as the intensity corresponding to a 50% predicted probability of blob formation, evaluated at the median mEGFP intensity within each condition. Confidence intervals were obtained by bootstrap resampling (1000 iterations), stratified by gephyrin condition.

### Confocal microscopy and image analysis

Image stacks [0.3-μm z-step size, 2520 × 2520 (144.77 × 144.77 μm)] were acquired on a Leica TCS SP8 LIGHTNING upright confocal microscope with an HC PL APO CS2 63×/1.30 glycerol objective, equipped with hybrid detectors (Leica HyD) and the following diode lasers: 405, 488, 552, and 638 nm. LIGHTNING adaptive deconvolution using “Mowiol” setting was applied. A random forest classifier was used for pixel classification (binarization) of clusters. Images were segmented and analyzed in an automated fashion using Python 3.10.8 (scikit-image 0.26.0, pyclesperanto-prototype 0.2.1, apoc 0.12.0, numpy 2.3.5, pandas 2.3.3, scipy 1.16.3, napari_simpleitk_image_processing, os, shutil 1.8.1), and the code is available online at GitHub (https://github.com/FilLieb/automated_synapse_analysis).

### Computation

No randomization procedure was performed when allocating samples to treatment. No samples were excluded from the analysis. Investigators were not blinded to sample allocation. Entire images from primary neuron cultures were analyzed in an automated fashion to minimize the effects of subjective bias. Statistical analyses for image analyses (analysis of variance (ANOVA) with Tukey’s post hoc test, Mann–Whitney U test) were performed using R version 4.5.2, rstatix v. 0.7.2, tidyverse v. 2.0.0, ggdist v. 3.3.3, distributional v. 0.7.0 in Rstudio environment (https://www.rstudio.com/), Platform: x86_64-w64-mingw32/x64. Data were tested for normality, using violation of the Shapiro–Wilk test at *p*  <  0.01 as the criterion. For all other statistical analyses GraphPad Prism 10 Version 10.2.3 was used for analysis of variance (ANOVA) with Tukey’s multiple comparisons test. Perplexity was used for language style improvement. OMERO, BioRender, and ChimeraX-1.8 were used for Fig. design.

## Supplementary information

Peer Review File

Source data Fig. 1

Source data Fig. 2

Source data Fig. 3

Source data Fig. 4

Source data Fig. 5

Source data Fig. 6

## Data Availability

This study includes no data deposited in external repositories. The source data of this paper are collected in the following database record: biostudies:S-SCDT-10_1038-S44321-026-00474-w.
