# Supplementary material for: Impaired gephyrin G-domain trimerization and phase separation in a patient with developmental epileptic encephalopathy
Source: EMBO Mol Med. 2026 Jun 30;18(8):3157–74. doi: 10.1038/s44321-026-00474-w (PMC13470343; doi:10.1038/s44321-026-00474-w)
Supplement: Supplementary file 1 [file 44321_2026_474_MOESM1_ESM.pdf]

EMBO Molecular Medicine

Impaired gephyrin G-domain trimerization and phase separation in a patient with developmental epileptic encephalopathy

Emanuel Bruckisch, Marcelo de Melo Aragão, Thais dos Santos Rhode, Ann-Kathrin Huber, Mateus de Oliveira Torres, Guenter Schwarz, and Filip Liebsch

Corresponding authors: Filip Liebsch (f.liebsch@uni-koeln.de) , Guenter Schwarz (gschwarz@uni-koeln.de)

Review Timeline:

Submission Date:	9th Jan 26
Editorial Decision:	9th Feb 26
Revision Received:	9th May 26
Editorial Decision:	21st May 26
Revision Received:	8th Jun 26
Accepted:	9th Jun 26

Editor: Jingyi Hou

Transaction Report:

(Note: With the exception of the correction of typographical or spelling errors that could be a source of ambiguity, letters and reports are not edited. Depending on transfer agreements, referee reports obtained elsewhere may or may not be included in this compilation. Referee reports are anonymous unless the Referee chooses to sign their reports.)

9th Feb 2026

Dear Dr. Liebsch,

Thank you for submitting your work to EMBO Molecular Medicine. We have now received evaluations from the two reviewers who agreed to assess your manuscript.

As you will see in the reports below, Reviewer #2 is generally supportive of the study, whereas Reviewer #1 has raised concerns regarding the depth of mechanistic insight, the strength of the genetic attribution, and certain aspects of the receptor-binding analysis. Both reviewers, however, have provided constructive suggestions on how these issues might be addressed.

As part of our pre-decision cross-commenting process (in which reviewers are given the opportunity to comment on each other's reports), both referees ultimately recommend a major revision. Reviewer #2 also provided specific feedback during this stage, which I have included below for your reference. Please consider these points when addressing the reviewers' comments.

We would welcome the submission of a revised version in three months for further consideration. As you may already know, our editorial policy allows in principle a single round of major revision, and it is therefore essential to provide responses to the referees' comments that are as complete as possible. Please feel free to contact me in case you would like to discuss in further detail any of the issues raised by the reviewers.

EMBO Molecular Medicine has a "scooping protection" policy, whereby similar findings that are published by others during review or revision are not a criterion for rejection. Should you decide to submit a revised version, I do ask that you get in touch after three months if you have not completed it, to update us on the status.

Please also contact us as soon as possible if similar work is published elsewhere. If other work is published, we may not be able to extend the revision period beyond three months.

Please read below for important editorial formatting and consult our author's guidelines for proper formatting of your revised article for EMBO Molecular Medicine.

I look forward to receiving your revised manuscript.

Sincerely,
Jingyi

Jingyi Hou
Senior Editor
EMBO Molecular Medicine

*****
When preparing your revised manuscript, please refer to our guidelines: https://link.springer.com/journal/44321/submission-guidelines#cms-Revised-submissions. We perform an initial quality control of all revised manuscripts before re-review; failure to include requested items will delay the evaluation of your revision.

We require:

1) A .docx formatted version of the manuscript text (including legends for main figures, EV figures and tables). Please make sure that the changes are highlighted to be clearly visible.

2) Individual production quality figure files as .eps, .tif, .jpg (one file per figure). For guidance, download the 'Figure Guide PDF': https://media.springernature.com/original/springer-cms/rest/v1/content/27825798/data/v1.

3) A .docx formatted letter INCLUDING the reviewers' reports and your detailed point-by-point responses to their comments. As part of the EMBO Press transparent editorial process, the point-by-point response is part of the Review Process File (RPF), which will be published alongside your paper.

4) A complete author checklist, which you can download from our author guidelines. Please insert information in the checklist that is also reflected in the manuscript. The completed author checklist will also be part of the RPF.

5) Please note that all corresponding authors are required to supply an ORCID ID for their name upon submission of a revised manuscript.

6) It is mandatory to include a 'Data Availability' section after the Materials and Methods. Before submitting your revision, primary datasets produced in this study need to be deposited in an appropriate public database, and the accession numbers and database listed under 'Data Availability'. Please remember to provide a reviewer password if the datasets are not yet public.

In case you have no data that requires deposition in a public database, please state so in this section. Note that the Data Availability Section is restricted to new primary data that are part of this study.

7) For data quantification: please specify the name of the statistical test used to generate error bars and P values, the number (n) of independent experiments (specify technical or biological replicates) underlying each data point and the test used to calculate p-values in each figure legend. The figure legends should contain a basic description of n, P and the test applied. Graphs must include a description of the bars and the error bars (s.d., s.e.m.).

8) At EMBO Press we ask authors to provide source data for the main manuscript figures. You will receive a separate email with instructions for providing source data with your revised manuscript, including how to upload and organize the files.

9) Our journal encourages inclusion of *data citations in the reference list* to directly cite datasets that were re-used and obtained from public databases. Data citations in the article text are distinct from normal bibliographical citations and should directly link to the database records from which the data can be accessed. In the main text, data citations are formatted as follows: "Data ref: Smith et al, 2001" or "Data ref: NCBI Sequence Read Archive PRJNA342805, 2017". In the Reference list, data citations must be labeled with "[DATASET]". A data reference must provide the database name, accession number/identifiers and a resolvable link to the landing page from which the data can be accessed at the end of the reference.

10) We replaced Supplementary Information with Expanded View (EV) Figures and Tables that are collapsible/expandable online. A maximum of 5 EV Figures can be typeset. EV Figures should be cited as 'Figure EV1, Figure EV2" etc... in the text and their respective legends should be included in the main text after the legends of regular figures.

- For the figures that you do NOT wish to display as Expanded View figures, they should be bundled together with their legends in a single PDF file called *Appendix*, which should start with a short Table of Content. Appendix figures should be referred to in the main text as: "Appendix Figure S1, Appendix Figure S2" etc.

- Additional Tables/Datasets should be labeled and referred to as Table EV1, Dataset EV1, etc. Legends have to be provided in a separate tab in case of .xls files. Alternatively, the legend can be supplied as a separate text file (README) and zipped together with the Table/Dataset file.

11) The paper explained: EMBO Molecular Medicine articles are accompanied by a summary of the articles to emphasize the major findings in the paper and their medical implications for the non-specialist reader. Please provide a draft summary of your article highlighting

- the medical issue you are addressing,

- the results obtained and

- their clinical impact.

This may be edited to ensure that readers understand the significance and context of the research. Please refer to any of our published articles for an example.

12) Author contributions: You will be asked to provide CRediT (Contributor Role Taxonomy) terms in the submission system. These replace a narrative author contribution section in the manuscript.

13) A Conflict of Interest statement should be provided in the main text.

14) Please provide a 'Synopsis' to further enhance discoverability. Synopses are displayed on the journal webpage and are freely accessible to all readers. They include a short stand first (maximum of 300 characters, including space) as well as 2-5 one-sentences bullet points that summarizes the paper. Please write the bullet points to summarize the key NEW findings. They should be designed to be complementary to the abstract - i.e. not repeat the same text. We encourage inclusion of key acronyms and quantitative information (maximum of 30 words / bullet point). Please use the passive voice. Please attach these

in a separate file or send them by email, we will incorporate them accordingly.

Please also suggest a visual abstract to illustrate your article as a PNG file 550 px wide x 300-600 px high.

15) Please download and fill our Reagents and Tools Table template (.docx), which you can find in our author guidelines: https://link.springer.com/journal/44320/submission-guidelines#structuredmethods.

EMBO Molecular Medicine has a "scooping protection" policy, whereby similar findings that are published by others during review or revision are not a criterion for rejection. Should you decide to submit a revised version, I do ask that you get in touch after three months if you have not completed it, to update us on the status.

***** Reviewer's comments *****

Referee #1 (Comments on Novelty/Model System for Author):

Aligning this study with the scope of EMBO Mol Med, it does "deepen our understanding of human disease" because it makes a new addition to quite a number of known Gph variants that cause epilepsy, some of which also affect LLPS. However, the new "mechanistic insight and translational implications in preclinical disease models and humans" are limited. The new Gph variant was discovered in a patient. The effect of the new variant on the formation of higher-order Gph assemblies and LLPS is surprising and interesting, but the underlying mechanism can only be speculated on. No experiments are provided to address this issue mechanistically. The study is restricted to in-vitro and cell culture experiments in cell lines and primary neurons. No preclinical disease models (e.g. in mice) were used to study the variant in the intact brain.

Referee #1 (Remarks for Author):

Bruckisch et al. present a clearly written and interesting account of a study on the characterization of a novel Gepyhrin (Gph) variant in a patient that likely causes epilepsy and developmental delay. Analysis of this variant shows that it leads to deficits in the formation of higher-order Gph oligomers and the formation of LLPS Gph, with corresponding consequences for Gph function in transmitter receptor clustering and Moco biosynthesis. The new G134R-Gph variant is a new addition to quite a number of known Gph variants that cause epilepsy, some of which also affect LLPS.

I have the following comments that I would like the authors to consider:

1. The genetic analysis is limited to a gene panel for epilepsy and metabolic disorders. This means that it cannot be formally excluded that another, as yet unidentified, gene variant contributes to the clinical phenotype of the patient. Whole exome or, even better, whole genome sequencing would help to resolve this uncertainty. This can be done via commercial services, if needed, without excessive cost.

2. As concerns the analysis of receptor binding to the Gph variant, I was suprised to see that GlyR binding (GlyR-beta-ICD peptide) was tested in the ITC experiments while the other experiments focused on GABA-A-Rs. Given the different Gph affinities of Gly-Rs and GABA-A-Rs, I wonder whether the effects of the G134R exchange on receptor binding can simply be extrapolated from Gly-Rs to GABA-A-Rs. Further, the effects of the G134R exchange on the formation of higher-order Gph assemblies and LLPS appear to be caused by a complex interplay between the main oligomerising domains and possibly conformational changes in the full-length protein. I therefore wonder whether binding assays with receptor proteins instead of synthetic peptides representing the minimal binding domain might be more informative.

3. Despite the authors' extensive and speculative discussion, it remains unclear how the G134R-variant affects Gph oligomerisation and LLPS. Sorting this out is clearly difficult and will require modeling and structural biology approaches. But the discussion of the issue is hard for me to penetrate. It might help to support the argumentation of the corresponding discussion section with schemata to guide the reader through the authors' reasoning.

Overall, this is an informative study concerned with a well-established topic. A number of Gph variants causing epilepsy are already known, some of which also affect Gph LLPS. New in the present study, in this context, is the discovery of an epilepsy-causing and LLPS-perturbing missense variant in the G-domain of Gph. The corresponding gain of information as regards the etiology of epilepsy and corresponding therapeutic approaches is interesting but does not represent a major step forward. Fascinating is the surprising - to me - effect of the new variant on the formation of higher-order Gph assemblies and LLPS, but the underlying mechanism can only be speculated on. Aligning this with the scope of EMBO Mol Med, the present study does "deepen our understanding of human disease" but the new "mechanistic insight and translational implications in preclinical disease models and humans" are limited in my view.

Referee #2 (Comments on Novelty/Model System for Author):

A de novo pathogenic mutation identified affecting the synaptic clustering protein gephyrin in a patient with developmental delay, epileptic seizures, microcephaly, dysmorphic features and short stature that disrupts higher-order oligomerization and Moco synthesis, leading to impaired liquid-liquid phase separation (LLPS) and synaptic clustering, acting in a domainant-negative manner. Sophisticated range of functional analyses of mutation impacts and novelty (first G domain mutation linked to functional deficits).

Referee #2 (Remarks for Author):

Bruckisch and colleagues describe a de novo pathogenic mutation identified affecting the synaptic clustering protein gephyrin in a patient with DEE. The G134R substitution disrupts higher-order gephyrin oligomerization, leading to impaired liquid-liquid phase separation (LLPS) and synaptic clustering. In non-neuronal cells, G134R forms dimers rather than trimers, and loses around 50% of Moco synthesis function. The study shows a sophisticated functional analysis of the impacts of the mutation G134R and novelty, i.e. the first gephyrin G domain mutation linked to a range of functional deficits, including LLPS and synaptic gephyrin clustering. Comments to be addressed by authors:

1. Abstract, line 21. Epileptic patient with developmental delay and seizures, might be better written as 'individual with developmental delay, epileptic seizures, microcephaly, dysmorphic features and short stature', i.e. give the full range of clinical phenotypes - and avoid the use of 'patient'. 'G134R fails to oligomerize beyond dimers and loses Moco synthesis function'. The loss of Moco activity should be quantified, i.e. around 50%? It might also be worth mentioning that binding to GlyR beta and GABA-AR alpha3 is unaffected.
2. Line 56 - Mutations in gephyrin can thus impair metabolic functions, leading to the fatal metabolic disorder Moco deficiency (MoCD). Given the de novo nature of this mutation, it would be useful to clarify the mutations causing Moco deficiency vs DEEs, neurodevelopmental and neuropsychiatric disorders, and how these differ in terms of gephyrin domains and/or inheritance. This could be in the form of a figure or table.
3. Line 95 - give details of the benzodiazepine used therapeutically.
4. Line 102 - have the authors checked the frequency of the c.400G>A; p.Gly134Arg variant in databases such as Gnomad? https://gnomad.broadinstitute.org/ I would suggest including this information, as it may support the assertion that this is a rare, de novo variant. I would also suggest adding a Combined Annotation Dependent Depletion (CADD) score for this mutation: https://cadd.gs.washington.edu/
5. Line 110 and figure 1D/1E. the molecular modelling shown in this figure is inadequate. Since structures of the human gephyrin G domain exist e.g. PDB 1EAV and the authors assert that p.G134R disrupts gephyrin G domain oligomerisation, the authors should study the predicted effects of this substitution e.g. using ChimeraX software (SwapAA command) or molecular dynamics simulations. It is hard to understand how replacing glycine with arginine to create a positively-charged surface residue could impact on G domain oligomerisation, and such analysis may provide some clues.
6. Line 159 - exact values with statistics (i.e. variation) should be given for relative Moco activity for G134R and G134R/WT mixes. The text states 50% for G134R, but from figure 3C the activity of G134R seems to be closer to 60%.
7. Line 187-196. There is no statistical analysis of gephyrin phase separation experiments - and significant results are not shown in figures 4C and F. This information must be provided and the figures should be amended.
8. Figure 5 - the images shown in figures 5D and E are inadequate - the entire cell is not in frame in many cases and at least two different cellular phenotypes are observable in figure 5E - single gephyrin blobs and multiple blobs. If different cellular phenotypes are observed, this should be quantified - and images with a few cells rather than single cropped cells should be shown. It is also noteworthy that the G134R mutant does not appear to form large perinuclear gephyrin blobs as is common for wild-type gephyrin. Have the authors checked whether dimeric controls (e.g. G2-gephyrin) or trimeric mutants (e.g. S505F) show similar subcellular localisation? This comparison would add strength to the arguments that the G134R mutant is influencing gephyrin oligomerisation.

****

Pre-decision cross-commenting

Referee #2

I concur that the authors should be given the chance to revise the manuscript.
Comments on referee 1 suggestions:

1. Genetic analysis with gene panel. this is very common in genetic analysis nowadays, and I would suggest the authors should mention this as a limitation of the analysis. They could also provide details on any other variants of interest that were identified, and why the gopher in variant was prioritised, e.g. CADD scores etc. I do not think that exome sequencing would add to the study.

2. Receptor binding. I do not understand this referees comment as the authors measure both GlyR and GABA-AR binding. Both

bind to the gopher in E domain and this is an important aspect of the analysis, i.e. that binding of both receptor types is unaffected. Binding assays with minimal binding domains are well established assays within the field.

3. Mechanism of G134R - I agree, and hope that this can be addressed by molecular modeling/moilecular dynamics.

I disagree that the paper is not a step forward. The authors have conducted assays for all major functions of gephyrin, Moco synthesis, receptor binding, oligomerisation and synaptic targeting. Preclinical disease models (e.g. knock-in animal models) are outside the scope of what can reasonably (and affordably) be done in the current functional study. KI lines take years to generate and characterise. However, I would point out that the authors do conduct studies in cultured neurones, so at least a relevant in vitro animal model is used.

Point-to-Point answers in green (also the adequate changes in the revised manuscript are in green)

Referee #1 (Comments on Novelty/Model System for Author):

Aligning this study with the scope of EMBO Mol Med, it does "deepen our understanding of human disease" because it makes a new addition to quite a number of known Gph variants that cause epilepsy, some of which also affect LLPS. However, the new "mechanistic insight and translational implications in preclinical disease models and humans" are limited. The new Gph variant was discovered in a patient. The effect of the new variant on the formation of higher-order Gph assemblies and LLPS is surprising and interesting, but the underlying mechanism can only be speculated on. No experiments are provided to address this issue mechanistically. The study is restricted to in-vitro and cell culture experiments in cell lines and primary neurons. No preclinical disease models (e.g. in mice) were used to study the variant in the intact brain.

ANSWER: We are grateful for the reviewer's thoughtful evaluation of our work and sincerely appreciate the recognition that our study deepens the understanding of human disease by adding a novel *GPHN* variant to the growing spectrum of epilepsy-associated mutations.

We also thank the reviewer for the constructive and critical perspective regarding mechanistic depth and translational scope. We respectfully refer here to the pre-decision cross-commenting by Reviewer #2, who stated: "I disagree that the paper is not a step forward. The authors have conducted assays for all major functions of gephyrin, Moco synthesis, receptor binding, oligomerisation and synaptic targeting. Preclinical disease models (e.g. knock-in animal models) are outside the scope of what can reasonably (and affordably) be done in the current functional study. KI lines take years to generate and characterise. However, I would point out that the authors do conduct studies in cultured neurones, so at least a relevant *in vitro* animal model is used."

We are grateful for this assessment, which we believe accurately reflects both the strengths and the realistic boundaries of the present study. Importantly, our experimental approach does not rely solely on heterologous cell lines. We performed analyses in primary mouse hippocampal neurons, thereby employing a physiologically relevant neuronal system. While these experiments are conducted *in vitro*, they allow us to assess synaptic targeting and clustering in a native neuronal context. We have clarified this point more explicitly in the revised manuscript discussion. We fully agree that the generation and characterization of a knock-in mouse model may provide additional physiological insight. However, such preclinical models are far beyond what can be achieved within the framework of a functional follow-up study of a single, unique patient-derived variant. The development of a knock-in line requires substantial time, financial resources, and long-term phenotypic characterization. While interesting, particularly given the rarity and uniqueness of this case, this would constitute a major independent research program rather than a feasible extension of the present work.

Regarding the mechanistic aspects of disrupted higher-order assembly and LLPS, we agree that our current data allow us to propose mechanistic explanations but do not yet fully resolve the structural basis at atomic resolution. Our intention was to comprehensively assess the major functional axes of gephyrin to establish whether and how the patient-derived variant perturbs gephyrin biology: Moco synthesis, receptor interaction, oligomerization, phase separation, and synaptic targeting. In this respect, we believe the study provides an integrative functional characterization that meaningfully advances understanding of the disease-associated mutation, even if certain mechanistic questions remain open for future structural or *in vivo* studies.

We have revised the Discussion to more clearly acknowledge these limitations while emphasizing the translational relevance of identifying and functionally validating a patient-derived variant that directly disrupts gephyrin assembly and synaptic organization.

Referee #1 (Remarks for Author):

Bruckisch et al. present a clearly written and interesting account of a study on the characterization of a novel Gepyhrin (Gph) variant in a patient that likely causes epilepsy and developmental delay. Analysis of this variant shows that it leads to deficits in the formation of higher-order Gph oligomers and the formation of LLPS Gph, with corresponding consequences for Gph function in transmitter receptor clustering and Moco biosynthesis. The new G134R-Gph variant is a new addition to quite a number of known Gph variants that cause epilepsy, some of which also affect LLPS.

I have the following comments that I would like the authors to consider:

1. The genetic analysis is limited to a gene panel for epilepsy and metabolic disorders. This means that it cannot be formally excluded that another, as yet unidentified, gene variant contributes to the clinical phenotype of the patient. Whole exome or, even better, whole genome sequencing would help to resolve this uncertainty. This can be done via commercial services, if needed, without excessive cost.

ANSWER: We thank the reviewer for this critical and important comment regarding the scope of the genetic analysis. We fully agree that the use of a targeted gene panel does not formally exclude the possibility that an additional, as yet unidentified, variant could contribute to the patient's clinical phenotype. As also pointed out by Reviewer 2 in the pre-decision cross-commenting ("Genetic analysis with gene panel: This is very common in genetic analysis nowadays, and I would suggest the authors should mention this as a limitation of the analysis. […] I do not think that exome sequencing would add to the study"), gene panel–based next-generation sequencing (NGS) is currently a widely used and clinically established diagnostic approach.

In response to both reviewers, we have revised the manuscript discussion part to more clearly state the inherent limitations of panel-based NGS, explicitly acknowledging that variants outside the captured regions cannot be excluded.
In addition, we now provide further details on another variant identified in the analysis and clarify why the gephyrin variant was prioritized. Specifically, we added the report on the additional intronic variant in *SCN9A* (encoding the sodium voltage-gated

channel alpha subunit 9) in the clinical description of the patient. This variant is classified as only “likely benign”, is located in a non-coding region, and was also detected in the clinically unaffected mother. Moreover, pathogenic variants in *SCN9A* are primarily associated with altered pain perception phenotypes rather than epilepsy or the complex neurodevelopmental presentation observed in our patient. These considerations argue against a relevant contribution of this variant to the phenotype.

To further strengthen the genetic interpretation, we have expanded the *in silico* analyses in the revised manuscript. This includes the addition of CADD scores and allele frequency data from the gnomAD database, supporting the predicted deleteriousness and rarity of the gephyrin variant. Together with the functional data presented, this provides a coherent line of evidence for its pathogenic relevance. Regarding whole exome or whole genome sequencing, while we acknowledge that broader sequencing approaches may theoretically reduce residual uncertainty, Reviewer 2 also concluded that exome sequencing would be unlikely to add substantially to the present study.

Given the clinical context, segregation data, *in silico* prediction, rarity in population databases, and the functional characterization performed, we believe the current level of genetic analysis is appropriate for the scope of this study.

We have revised the manuscript accordingly to clarify these points and to transparently discuss the limitations of the genetic approach.

2. As concerns the analysis of receptor binding to the Gph variant, I was suprised to see that GlyR binding (GlyR-beta-ICD peptide) was tested in the ITC experiments while the other experiments focused on GABA-A-Rs. Given the different Gph affinities of Gly-Rs and GABA-A-Rs, I wonder whether the effects of the G134R exchange on receptor binding can simply be extrapolated from Gly-Rs to GABA-A-Rs. Further, the effects of the G134R exchange on the formation of higher-order Gph assemblies and LLPS appear to be caused by a complex interplay between the main oligomerising domains and possibly conformational changes in the full-length protein. I therefore wonder whether binding assays with receptor proteins instead of synthetic peptides representing the minimal binding domain might be more informative.

ANSWER: We thank the reviewer for this insightful and technically very relevant comment regarding receptor binding assays and the choice of model systems. We appreciate the opportunity to clarify our rationale. Structural and biochemical studies have firmly established that GlyR β and synaptic $GABA_A$ receptor α1–3 subunits bind via homologous TM3–TM4 intracellular loop motifs to a common receptor-binding pocket within the gephyrin E-domain, centered on the conserved Phe330 epitope. This has been demonstrated in detail (Maric *et al.*, 2014) and reviewed comprehensively (Tyagarajan and Fritschy, 2014). These studies provide strong evidence that the molecular recognition mechanism is highly conserved between GlyRs and major synaptic $GABA_A$ receptor subtypes, supporting the use of GlyR β-derived peptides as a structurally validated proxy for gephyrin receptor binding. In line with this established framework, the GlyR β intracellular domain (ICD) peptide has become the standard model ligand for quantitative ITC-based binding studies in the field.

Importantly, in previously published functional analyses of single patient–derived gephyrin variants, the GlyR model peptide was consistently used for biophysical binding assays, allowing direct comparison across studies. We therefore deliberately employed this well-characterized and field-accepted model system to ensure methodological robustness and comparability. We would also like to refer to the pre-decision cross-commenting by Reviewer #2, who noted that binding assays using minimal binding domains are well-established assays within the field because they isolate the direct interaction interface and enable precise thermodynamic quantification, which would be significantly more challenging with full-length, membrane-embedded receptor complexes.

Regarding the concern that receptor binding behavior might not be directly extrapolated between GlyRs and $GABA_A$Rs, we would like to emphasize that our LLPS experiments were not restricted to a single model. All *in vitro* LLPS assays were performed using a GlyR model as well as two distinct $GABA_A$ receptor-derived models. As also highlighted by Reviewer #2 in the cross-commenting, both model systems exhibited similar behavior with respect to LLPS in the low-oligomer fraction. This supports the notion that the observed effects of the G134R variant on higher-order assembly and phase separation are not specific to a single receptor model but reflect a broader perturbation of gephyrin scaffold function.

Finally, while we agree that assays with full-length receptor proteins could in principle provide additional layers of information, such experiments are technically highly demanding due to the membrane-bound and multimeric nature of these receptors. Within the scope of a mechanistic functional characterization study, we therefore relied on structurally validated minimal binding motifs and multiple receptor-derived models to achieve a balance between biochemical precision, technical feasibility, and physiological relevance.

3. Despite the authors' extensive and speculative discussion, it remains unclear how the G134R-variant affects Gph oligomerisation and LLPS. Sorting this out is clearly difficult and will require modeling and structural biology approaches. But the discussion of the issue is hard for me to penetrate. It might help to support the argumentation of the corresponding discussion section with schemata to guide the reader through the authors' reasoning.

ANSWER: We appreciate this thoughtful suggestion. We agree that the conceptual framework underlying our hypothesis can be challenging to follow purely in text form, and that a schematic representation can substantially improve accessibility and clarity. We also note that reviewer #2 made a similar recommendation, which further motivated us to strengthen this aspect of the manuscript.

In response, we have added a new Figure 7 to visually support and structure the reasoning presented in the discussion.
In this schematic, we differentiate between:
- Center: A highly percolated WT gephyrin network, which we hypothesize is required for efficient receptor immobilization and stable synaptic clustering.
- Left: Previously published E-domain variants affecting subdomain II (G375D and D422N), which lead to loss of filament formation and impaired LLPS, as reported in the literature.

- Right: The newly identified G134R variant, which we propose disrupts G-domain–mediated interconnectivity necessary for network percolation, thereby reducing higher-order assembly and LLPS.

We believe this visual summary substantially improves the clarity of the mechanistic argumentation and helps guide the reader through the proposed model.

Overall, this is an informative study concerned with a well-established topic. A number of Gph variants causing epilepsy are already known, some of which also affect Gph LLPS. New in the present study, in this context, is the discovery of an epilepsy-causing and LLPS-perturbing missense variant in the G-domain of Gph. The corresponding gain of information as regards the etiology of epilepsy and corresponding therapeutic approaches is interesting but does not represent a major step forward. Fascinating is the surprising - to me - effect of the new variant on the formation of higher-order Gph assemblies and LLPS, but the underlying mechanism can only be speculated on. Aligning this with the scope of EMBO Mol Med, the present study does "deepen our understanding of human disease" but the new "mechanistic insight and translational implications in preclinical disease models and humans" are limited in my view.

ANSWER: We sincerely appreciate the reviewer's balanced and thoughtful overall assessment of our study. We are grateful for the recognition that our work deepens the understanding of human disease and that the identification of a G-domain missense variant affecting higher-order assembly and LLPS is considered surprising and interesting. We fully acknowledge the reviewer's perspective regarding the degree of mechanistic depth and translational scope. At the same time, we would like to emphasize in line with the pre-decision cross-commenting by Reviewer #2, that this study represents a comprehensive functional analysis of a patient-derived *GPHN* variant. As Reviewer #2 stated: "I disagree that the paper is not a step forward. The authors have conducted assays for all major functions of gephyrin, Moco synthesis, receptor binding, oligomerisation and synaptic targeting. Preclinical disease models (e.g. knock-in animal models) are outside the scope of what can reasonably (and affordably) be done in the current functional study."

We believe this accurately reflects the integrative nature of our approach. Rather than focusing on a single biochemical readout, we systematically assessed all principal functional axes of gephyrin biology, thereby providing a broad and coherent characterization of the variant. With respect to translational implications, we would like to clarify that the translational aspect of our study lies less in immediate therapeutic modeling and more in defining a novel molecular node within the gephyrin scaffold that is vulnerable to pathogenic perturbation. By identifying a G-domain missense mutation that disrupts higher-order assembly and LLPS, we expand the mechanistic landscape of gephyrin-associated epilepsies beyond previously characterized regions. In this sense, our work delineates a new structural-functional hotspot within the scaffold, thereby refining the conceptual framework for how single patient–derived variants can impair inhibitory synapse organization. This provides an important foundation for future precision-medicine approaches and for mechanistic stratification of gephyrin-related epilepsies in subsequent patient cohorts or experimental models.

Importantly, there is also a clinically relevant dimension: the patient responded to benzodiazepine treatment. Benzodiazepines increase the opening frequency of $GABA_A$ receptors, thereby enhancing inhibitory signaling even when receptor clustering is reduced. In the context of our findings, this pharmacological mechanism is conceptually consistent with the clinical improvement observed following benzodiazepine treatment. Thus, although indirect, the molecular insights gained here are aligned with therapeutic responsiveness at the patient level.

We agree that certain mechanistic aspects, particularly the structural basis of altered higher-order assembly, remain to be resolved in future studies. Nevertheless, we believe that the identification and integrative functional validation of a unique, patient-derived G-domain variant constitutes a meaningful advance in the field and provides a solid basis for further mechanistic and translational investigations.

Referee #2 (Comments on Novelty/Model System for Author):

A de novo pathogenic mutation identified affecting the synaptic clustering protein gephyrin in a patient with developmental delay, epileptic seizures, microcephaly, dysmorphic features and short stature that disrupts higher-order oligomerization and Moco synthesis, leading to impaired liquid-liquid phase separation (LLPS) and synaptic clustering, acting in a domainant-negative manner. Sophisticated range of functional analyses of mutation impacts and novelty (first G domain mutation linked to functional deficits).

Referee #2 (Remarks for Author):

Bruckisch and colleagues describe a de novo pathogenic mutation identified affecting the synaptic clustering protein gephyrin in a patient with DEE. The G134R substitution disrupts higher-order gephyrin oligomerization, leading to impaired liquid-liquid phase separation (LLPS) and synaptic clustering. In non-neuronal cells, G134R forms dimers rather than trimers, and loses around 50% of Moco synthesis function. The study shows a sophisticated functional analysis of the impacts of the mutation G134R and novelty, i.e. the first gephyrin G domain mutation linked to a range of functional deficits, including LLPS and synaptic gephyrin clustering. Comments to be addressed by authors:

1. Abstract, line 21. Epileptic patient with developmental delay and seizures, might be better written as 'individual with developmental delay, epileptic seizures, microcephaly, dysmorphic features and short stature', i.e. give the full range of clinical phenotypes - and avoid the use of 'patient'. 'G134R fails to oligomerize beyond dimers and loses Moco synthesis function'. The loss of Moco activity should be quantified, i.e. around 50%? It might also be worth mentioning that binding to GlyR beta and GABA-AR alpha3 is unaffected.

ANSWER: We thank the reviewer for these constructive and precise suggestions regarding the abstract. We agree that the proposed modifications improve clarity and completeness.

- We have revised the wording to describe the full clinical spectrum, now referring to an individual with developmental delay, epileptic seizures, microcephaly, dysmorphic

features and short stature, thereby avoiding the term “patient” and providing a more comprehensive clinical description.
- The reduction in Moco synthesis activity has now been quantified in the abstract
- We have additionally clarified that binding to GlyR β and $GABA_AR$ α3 is unaffected, in order to present a balanced summary of the functional findings.

We appreciate the reviewer’s attention to these details, which have strengthened the clarity and precision of the abstract.

2. Line 56 - Mutations in gephyrin can thus impair metabolic functions, leading to the fatal metabolic disorder Moco deficiency (MoCD). Given the de novo nature of this mutation, it would be useful to clarify the mutations causing Moco deficiency vs DEEs, neurodevelopmental and neuropsychiatric disorders, and how these differ in terms of gephyrin domains and/or inheritance. This could be in the form of a figure or table.

ANSWER: We appreciate this insightful suggestion, as it highlights an important conceptual distinction within the spectrum of gephyrin-associated disorders. Clarifying the differences between variants causing Moco deficiency (MoCD) and those linked to developmental and epileptic encephalopathies (DEEs), neurodevelopmental, or neuropsychiatric conditions is particularly relevant given the *de novo* nature of the variant described in our study.

To address this point, we have expanded Figure 1A to explicitly distinguish variants implicated in epilepsy, neurodevelopmental phenotypes and Moco deficiency type C thereby providing a clearer overview of domain localization and functional context. This visual representation helps to delineate differences in mutational clustering and associated clinical outcomes. In addition, we have revised and extended the corresponding section in the main text.

We believe these additions substantially improve the clarity, mechanistic understanding, and clinical framing of the manuscript.

3. Line 95 - give details of the benzodiazepine used therapeutically.

ANSWER: This is an important point, and we agree that specifying the benzodiazepine treatment improves clinical clarity.

We have now added the relevant therapeutic details to the manuscript: the individual underwent trials with topiramate, valproate, lamotrigine, and levetiracetam. The patient is currently treated with 10 mg clobazam, has maintained good seizure control, and no further events have been reported, including normalization of the EEG. We believe that including the specific benzodiazepine treatment strengthens the translational aspect of our study by directly linking the molecular findings to therapeutic response.

4. Line 102 - have the authors checked the frequency of the c.400G>A; p.Gly134Arg variant in databases such as Gnomad? https://gnomad.broadinstitute.org/ I would suggest including this information, as it may support the assertion that this is a rare, de novo variant. I would also suggest adding a Combined Annotation Dependent Depletion (CADD) score for this mutation: https://cadd.gs.washington.edu/

ANSWER: We are particularly grateful for this excellent suggestion, as incorporating these analyses substantially strengthens the genetic interpretation of our study.

Following the reviewer's recommendation, we have systematically checked both gnomAD and CADD and have included the respective information in the revised manuscript. In gnomAD, the variant (gnomAD variant ID: 14-66916013-G-A) is only listed when filtered variants that do not pass the full quality control process are included, and it appears only once. Under standard high-quality filtering criteria, the allele count is zero. This strongly supports the rarity of the c.400G>A; p.Gly134Arg variant and is consistent with its *de novo* occurrence. In addition, we have included the CADD score for this variant. The CADD score of 33.0 places it among the top 0.05% most deleterious potential substitutions in the human genome, further supporting its predicted pathogenicity.

We thank the reviewer for this constructive recommendation, which has meaningfully reinforced the genetic evidence presented in our manuscript.

5. Line 110 and figure 1D/1E. the molecular modelling shown in this figure is inadequate. Since structures of the human gephyrin G domain exist e.g. PDB 1EAV and the authors assert that p.G134R disrupts gephyrin G domain oligomerisation, the authors should study the predicted effects of this substitution e.g. using ChimeraX software (SwapAA command) or molecular dynamics simulations. It is hard to understand how replacing glycine with arginine to create a positively-charged surface residue could impact on G domain oligomerisation, and such analysis may provide some clues.

ANSWER: We thank the reviewer for this detailed and technically informed comment, and we appreciate the opportunity to clarify our structural analysis strategy.

First, the structure shown in Figure 1C corresponds to a published crystal structure of the human gephyrin G-domain (PDB code: 1JLJ). We realize that this was not sufficiently clear in the original version of the manuscript text and was only specified in the figure legend. Now the PDB code (1JLJ) and the reference to the published crystal structure are additionally included in the main text to improve clarity and readability.

Second, the structure shown in Figure 1D represents a deep learning enhanced protein structure prediction generated using the AlphaFold3 algorithm (Jumper *et al.*, 2021). This approach allows modeling of structural consequences of amino acid substitutions in the context of the entire protein and currently represents one of the most advanced prediction tools available short of extensive molecular dynamics (MD) simulations. We have included a more detailed description of the structural modeling workflow in the Materials and Methods section to ensure transparency.

With respect to the suggestion to use the SwapAA command in ChimeraX: this function performs a side-chain replacement without recalculating conformational adjustments of the surrounding structure or modeling induced structural rearrangements. While useful for visualization, it does not account for broader structural or energetic consequences of a substitution and therefore would not

provide additional mechanistic insight beyond what is already captured in our AlphaFold-based modeling.

Regarding the potential impact of replacing glycine with arginine, from our perspective, the introduction of a positively charged arginine residue at a position that contributes to a negatively charged surface region of the wild-type protein provides a plausible mechanistic explanation for disturbed intermolecular interactions within the G-domain trimer.

This altered electrostatic landscape is visualized in Figure 1E. To further strengthen this point and to address the reviewer's suggestion of more quantitative structural assessment, while acknowledging that full MD simulations are beyond the scope of our technical and computational resources, we additionally employed the web server DDMut-PPI to predict mutation-induced changes in protein–protein interaction stability. Specifically, we calculated the predicted change in binding free energy ($\Delta\Delta G$Binding) for G-domain trimerization. This analysis yielded a decreased affinity value of −2.53 ± 0.3 kcal/mol, consistent with destabilization of the trimeric assembly.

We have incorporated these clarifications and the additional computational analysis into the revised manuscript.

6. Line 159 - exact values with statistics (i.e. variation) should be given for relative Moco activity for G134R and G134R/WT mixes. The text states 50% for G134R, but from figure 3C the activity of G134R seems to be closer to 60%.

ANSWER: We appreciate the reviewer's careful evaluation of the quantitative data. In response, we have revised the manuscript to report the exact measured values instead of rounded estimates. The relative Moco synthesis activity of G134R-gephyrin is now stated as 64 ± 9%, consistent with the values shown in Figure 3C. We have also ensured that the corresponding mixed G134R/WT data are reported with precise values and associated variation (105 ± 3%).

We thank the reviewer for pointing out this discrepancy, which has improved the accuracy and consistency of the manuscript.

7. Line 187-196. There is no statistical analysis of gephyrin phase separation experiments - and significant results are not shown in figures 4C and F. This information must be provided and the figures should be amended.

ANSWER: We thank the reviewer for highlighting this important point regarding the statistical presentation of the phase separation experiments. In response, we have fused Figures 4C and 4F into a single, amended Figure 4C to clearly show the significant differences between high- and low-oligomer fractions of WT gephyrin for both the GlyR and $GABA_AR$-α3 models. A dashed line now separates the high and low oligomer fractions, and the G134R variant is shown only in the low-oligomer fraction using the consistent red color throughout the manuscript for clarity.

In the main text, we have clarified all references to high- or low-oligomer fractions by explicitly adding the words "High" or "Low" when referring to Figure 4C. Furthermore, statistical significance has been included, and all reported values have been updated

to exact numbers with associated variation. We believe these revisions improve both the clarity and rigor of the presentation of the LLPS data.

8. Figure 5 - the images shown in figures 5D and E are inadequate - the entire cell is not in frame in many cases and at least two different cellular phenotypes are observable in figure 5E - single gephyrin blobs and multiple blobs. If different cellular phenotypes are observed, this should be quantified - and images with a few cells rather than single cropped cells should be shown. It is also noteworthy that the G134R mutant does not appear to form large perinuclear gephyrin blobs as is common for wild-type gephyrin. Have the authors checked whether dimeric controls (e.g. G2-gephyrin) or trimeric mutants (e.g. S505F) show similar subcellular localisation? This comparison would add strength to the arguments that the G134R mutant is influencing gephyrin oligomerisation.

ANSWER: We thank the reviewer for this helpful suggestion and for highlighting the need for a more comprehensive and quantitative analysis.

To address these points, we acquired new datasets using live-cell imaging of HEK293 gephyrin knockout cells, thereby avoiding potential artefacts associated with fixation (Irgen-Gioro *et al.*, 2022). In addition, we expanded our experimental design to include variants that specifically disrupt G-domain trimerization (G2) and E-domain dimerization (S505F), as suggested. Representative images from these experiments, now showing cells in frame, are presented in Figure 5D,F.

To quantitatively assess the observed phenotypes, we measured cytosolic fluorescence intensities of gephyrin and GCN4-GlyR in more than 100 cells per condition across three independent experiments. These data are presented in Figure 5E,G. Consistent with previous reports (Lee *et al.*, 2024), blob formation occurred in a concentration-dependent manner. Importantly, both G-domain trimerization–deficient variants (G2 and G134R) and the E-domain dimerization–deficient variant (S505F) exhibited a shift in blob formation to higher cytosolic gephyrin concentrations, indicating reduced propensity for condensate formation. These findings agree with earlier studies using GFP-tagged gephyrin and related oligomerization-deficient mutants (Saiyed *et al.*, 2007).
The reviewer also noted the presence of distinct cellular phenotypes (single versus multiple blobs). Rather than discretely classifying these phenotypes, we quantified blob formation as a function of cytosolic gephyrin concentration. This approach captures the continuous and concentration-dependent nature of condensate formation.

In the presence of the dimeric receptor model GCN4-GlyR, we observed enhanced blob formation for WT gephyrin, consistent with previous findings (Lee et al., PNAS 2024). In contrast, disruption of G-domain trimerization (G2 and G134R) markedly impaired this effect, as reflected by a substantial increase in the concentration required for blob formation. Notably, disruption of E-domain dimerization (S505F) did not prevent this response; instead, GCN4-GlyR co-expression restored blob formation to levels comparable to WT highlighting the “cheparone”-like function of GNC4-GlyR in stabilizing the GephE dimer even under conditions of mutational reduction of dimer stability.

Together, these results support the conclusion that G-domain trimerization is critical for gephyrin condensation, whereas E-domain dimerization may be even dispensable in the presence of receptor scaffolding.

****

Pre-decision cross-commenting

Referee #2

I concur that the authors should be given the chance to revise the manuscript. Comments on referee 1 suggestions:

1. Genetic analysis with gene panel. this is very common in genetic analysis nowadays, and I would suggest the authors should mention this as a limitation of the analysis. They could also provide details on any other variants of interest that were identified, and why the gopher in variant was prioritised, e.g. CADD scores etc. I do not think that exome sequencing would add to the study.

2. Receptor binding. I do not understand this referees comment as the authors measure both GlyR and GABA-AR binding. Both bind to the gopher in E domain and this is an important aspect of the analysis, i.e. that binding of both receptor types is unaffected. Binding assays with minimal binding domains are well established assays within the field.

3. Mechanism of G134R - I agree, and hope that this can be addressed by molecular modeling/moilecular dynamics.

I disagree that the paper is not a step forward. The authors have conducted assays for all major functions of gephyrin, Moco synthesis, receptor binding, oligomerisation and synaptic targeting. Preclinical disease models (e.g. knock-in animal models) are outside the scope of what can reasonably (and affordably) be done in the current functional study. KI lines take years to generate and characterise. However, I would point out that the authors do conduct studies in cultured neurones, so at least a relevant in vitro animal model is used.

ANSWER: We are very grateful to Reviewer #2 for taking the opportunity to provide pre-decision cross-commenting. We consider this a valuable contribution to the revision process, and the insight provided have been extremely helpful in guiding improvements and clarifications throughout the manuscript.

REFERENCES:

Irgen-Gioro, S. *et al.* (2022) "Fixation can change the appearance of phase separation in living cells," *eLife*. Edited by F. Campelo et al., 11, p. e79903. Available at: https://doi.org/10.7554/eLife.79903.

Jumper, J. *et al.* (2021) "Highly accurate protein structure prediction with AlphaFold," *Nature*, 596(7873), pp. 583–589. Available at: https://doi.org/10.1038/s41586-021-03819-2.

Lee, G. *et al.* (2024) "Thermodynamic modulation of gephyrin condensation by inhibitory synapse components," *Proceedings of the National Academy of Sciences*, 121(12), p. e2313236121. Available at: https://doi.org/10.1073/pnas.2313236121.

Maric, H.M. *et al.* (2014) "Molecular basis of the alternative recruitment of GABAA versus glycine receptors through gephyrin," *Nature Communications*, 5(1), p. 5767. Available at: https://doi.org/10.1038/ncomms6767.

Saiyed, T. *et al.* (2007) "Molecular basis of gephyrin clustering at inhibitory synapses: role of G-and E-domain interactions," *Journal of Biological Chemistry*, 282(8), pp. 5625–5632.

Tyagarajan, S.K. and Fritschy, J.-M. (2014) "Gephyrin: a master regulator of neuronal function?," *Nature Reviews Neuroscience*, 15(3), pp. 141–156.

21st May 2026

Dear Dr. Liebsch,

Thank you for the submission of your revised manuscript to EMBO Molecular Medicine. We have now received the enclosed report from the referee who was asked to re-assess it. As you will see, the referee is now supportive, and I am pleased to inform you that we will be able to accept your manuscript pending the following editorial-level amendments:

1. Please move "The paper explained" to the manuscript file and incorporate the subheadings "Problem", "Results", and "Impact".

2. Data availability : please change the current statement to" This study includes no data deposited in external repositories".

3. For the conflict of interest statement, please add a separate section with the section heading " Disclosure and Competeing Interests Statement".

4. "LITERATURE" should be renamed "REFERENCES". "et al." should be used after 10 author names. DOIs should only be included for preprints and datasets that have not yet been published.

5. Please remove the separate "Ethics Statement" section. The relevant information should instead be incorporated into the appropriate sections of the Methods.

6. "MATERIAL AND METHODS" should be renamed to "Methods".

7. Figure legends should be placed after References.

8. Please address the following issues in the figure legends:
- Please note that the legends for figures 4-6 is not provided in the sequential manner. This needs to be rectified.
- Please define the annotated p values ****/***/**/* as well as provide the exact p-values for the same in the legend of figure 6C, E as appropriate.
- Please note that the exact p values are not provided in the legends of figures 3C, 4C
- Please note that the measure of center for the error bars needs to be defined in the legend of figure 5E

I look forward to reading a new revised version of your manuscript as soon as possible.

Kind regards,
Jingyi

Jingyi Hou
Senior Editor
EMBO Molecular Medicine

*** Instructions to submit your revised manuscript ***

*** PLEASE NOTE *** As part of the EMBO Publications transparent editorial process initiative (see our Editorial at https://www.embopress.org/doi/pdf/10.1002/emmm.201000094), EMBO Molecular Medicine will publish online a Review Process File to accompany accepted manuscripts.

In the event of acceptance, this file will be published in conjunction with your paper and will include the anonymous referee reports, your point-by-point response and all pertinent correspondence relating to the manuscript. If you do NOT want this file to be published, please inform the editorial office at contact@embomolmed.org.

***** Reviewer's comments *****

Referee #2 (Comments on Novelty/Model System for Author):

A de novo pathogenic mutation p.G134R was identified affecting the synaptic clustering protein gephyrin in a patient with developmental delay, epileptic seizures, microcephaly, dysmorphic features and short stature that disrupts higher-order oligomerization and Moco synthesis, leading to impaired liquid-liquid phase separation (LLPS) and synaptic clustering, acting in a domainant-negative manner. A sophisticated range of functional analyses is used to analyse the mutation impacts. The finding has novelty in that it is the first gephyrin G domain mutation linked to this range of symptoms.

Referee #2 (Remarks for Author):

The authors have carefully considered the feedback from both referees and made changes to the manuscript that have substantially improved the manuscript. Responses to critique are clearly laid out in the point-by-point reply. In particular, new live cell staining analysis of gephyrin microaggregrates in gephyrin KO cells support the conclusion that G-domain trimerization is critical for gephyrin condensation. The addition of Figure 7 is also useful in terms of understanding the contribution of the gephyrin G domain to LLPS. I congratulate the authors on this important study.

The authors addressed the remaining editorial issues.

9th Jun 2026

Dear Dr. Liebsch,

We are pleased to inform you that your manuscript is accepted for publication and is now being sent to our publisher to be included in the next available issue of EMBO Molecular Medicine.

Your manuscript will be processed for publication by EMBO Press. It will be copy edited and you will receive page proofs prior to publication. Please note that you will be contacted by Springer Nature Author Services to complete licensing and payment information.

You may qualify for financial assistance for your publication charges - either via a Springer Nature fully open access agreement or an EMBO initiative. Check your eligibility: https://link.springer.com/journal/44321/how-to-publish-with-us

Should you be planning a Press Release on your article, please notify the production team via this form in order to coordinate publication and release dates: https://forms.cloud.microsoft/e/eH4A8JVP6g

If you have any questions, please do not hesitate to contact the Editorial Office. Thank you for your contribution to EMBO Molecular Medicine.

Yours sincerely,
Jingyi

Jingyi Hou
Senior Editor
EMBO Molecular Medicine

>>> Please note that it is EMBO Molecular Medicine policy for the transcript of the editorial process (containing referee reports and your response letter) to be published as an online supplement to each paper. If you do NOT want this, you will need to inform the Editorial Office via email immediately. More information is available here: https://link.springer.com/partners/embo-press/editorial-policies#Peer%20review

EMBO Press Author Checklist

Corresponding Author Name: Dr. Filip Liebsch	
Journal Submitted to: EMBO Molecular Medicine	
Manuscript Number: EMM-2026-23276	

USEFUL LINKS FOR COMPLETING THIS FORM
The EMBO Journal - Author Guidelines
EMBO Reports - Author Guidelines
Molecular Systems Biology - Author Guidelines
EMBO Molecular Medicine - Author Guidelines

Reporting Checklist for Life Science Articles (updated January
This checklist is adapted from Materials Design Analysis Reporting (MDAR) Checklist for Authors. MDAR establishes a minimum set of requirements in transparent reporting in the life sciences (see Statement of Task: 10.31222/osf.io/9sm4x). Please follow the journal's guidelines in preparing your
Please note that a copy of this checklist will be published alongside your article.

Abridged guidelines for figures
1. Data
The data shown in figures should satisfy the following conditions:
- the data were obtained and processed according to the field's best practice and are presented to reflect the results of the experiments in an accurate and unbiased manner.
- ideally, figure panels should include only measurements that are directly comparable to each other and obtained with the same assay.
- plots include clearly labeled error bars for independent experiments and sample sizes. Unless justified, error bars should not be shown for technical
- if n<5, the individual data points from each experiment should be plotted. Any statistical test employed should be justified.
- Source Data should be included to report the data underlying figures according to the guidelines set out in the authorship guidelines on Data

2. Captions
Each figure caption should contain the following information, for each panel where they are relevant:
- a specification of the experimental system investigated (eg cell line, species name).
- the assay(s) and method(s) used to carry out the reported observations and measurements.
- an explicit mention of the biological and chemical entity(ies) that are being measured.
- an explicit mention of the biological and chemical entity(ies) that are altered/varied/perturbed in a controlled manner.
- the exact sample size (n) for each experimental group/condition, given as a number, not a range;
- a description of the sample collection allowing the reader to understand whether the samples represent technical or biological replicates (including how many animals, litters, cultures, etc.).
- a statement of how many times the experiment shown was independently replicated in the laboratory.
- definitions of statistical methods and measures:
  - common tests, such as t-test (please specify whether paired vs. unpaired), simple χ2 tests, Wilcoxon and Mann-Whitney tests, can be unambiguously identified by name only, but more complex techniques should be described in the methods section;
  - are tests one-sided or two-sided?
  - are there adjustments for multiple comparisons?
  - exact statistical test results, e.g., P values = x but not P values < x;
  - definition of 'center values' as median or average;
  - definition of error bars as s.d. or s.e.m.

Please complete ALL of the questions below.
Select "Not Applicable" only when the requested information is not relevant for your study.

Materials

Newly Created Materials	Information included in the manuscript?	In which section is the information available? (Reagents and Tools Table, Materials and Methods, Figures, Data Availability Section)
New materials and reagents need to be available; do any restrictions apply?	Yes	Reagents and Tools Table, Material and Methods

Antibodies	Information included in the manuscript?	In which section is the information available? (Reagents and Tools Table, Materials and Methods, Figures, Data Availability Section)
For **antibodies** provide the following information:<br>- Commercial antibodies: RRID (if possible) or supplier name, catalogue number and or/clone number<br>- Non-commercial: RRID or citation	Yes	Reagents and Tools Table, Material and Methods

DNA and RNA sequences	Information included in the manuscript?	In which section is the information available? (Reagents and Tools Table, Materials and Methods, Figures, Data Availability Section)
**Short novel DNA or RNA including primers, probes**: provide the sequences.	Yes	Reagents and Tools Table, Material and Methods

Cell materials	Information included in the manuscript?	In which section is the information available? (Reagents and Tools Table, Materials and Methods, Figures, Data Availability Section)
**Cell lines:** Provide species information, strain. Provide accession number in repository **OR** supplier name, catalog number, clone number, and/**OR** RRID.	Yes	Reagents and Tools Table, Material and Methods
**Primary cultures:** Provide species, strain, sex of origin, genetic modification status.	Yes	Reagents and Tools Table, Material and Methods
Report if the cell lines were recently **authenticated** (e.g., by STR profiling) and tested for mycoplasma contamination.	Yes	Material and Methods

Experimental animals	Information included in the manuscript?	In which section is the information available? (Reagents and Tools Table, Materials and Methods, Figures, Data Availability Section)
**Laboratory animals or Model organisms:** Provide species, strain, sex, age, genetic modification status. Provide accession number in repository **OR** supplier name, catalog number, clone number, **OR** RRID.	Yes	Reagents and Tools Table, Material and Methods
**Animal observed in or captured from the field:** Provide species, sex, and age where possible.	Not Applicable	
Please detail **housing and husbandry conditions**.	Yes	Material and Methods

Plants and microbes	Information included in the manuscript?	In which section is the information available? (Reagents and Tools Table, Materials and Methods, Figures, Data Availability Section)
**Plants:** provide species and strain, ecotype and cultivar where relevant, unique accession number if available, and source (including location for collected wild specimens).	Not Applicable	
**Microbes:** provide species and strain, unique accession number if available, and source.	Yes	Reagents and Tools Table, Material and Methods

Human research participants	Information included in the manuscript?	In which section is the information available? (Reagents and Tools Table, Materials and Methods, Figures, Data Availability Section)
If collected and within the bounds of privacy constraints report on age, sex and gender or ethnicity for all study participants.	Yes	Material and Methods

Core facilities	Information included in the manuscript?	In which section is the information available? (Reagents and Tools Table, Materials and Methods, Figures, Data Availability Section)
If your work benefited from core facilities, was their service mentioned in the acknowledgments section?	Yes	Acknowledgements

Design

Study protocol	Information included in the manuscript?	In which section is the information available? (Reagents and Tools Table, Materials and Methods, Figures, Data Availability Section)
If study protocol has been **pre-registered, provide DOI in the manuscript**. For clinical trials, provide the trial registration number **OR** cite DOI.	Not Applicable	
Report the **clinical trial registration number** (at ClinicalTrials.gov or equivalent), where applicable.	Not Applicable	

Laboratory protocol	Information included in the manuscript?	In which section is the information available? (Reagents and Tools Table, Materials and Methods, Figures, Data Availability Section)
Provide DOI OR other citation details if **external detailed step-by-step protocols** are available.	Not Applicable	

Experimental study design and statistics	Information included in the manuscript?	In which section is the information available? (Reagents and Tools Table, Materials and Methods, Figures, Data Availability Section)
Include a statement about **sample size** estimate even if no statistical methods were used.	Yes	Materials and Methods, Figures
Were any steps taken to minimize the effects of subjective bias when allocating animals/samples to treatment (e.g. **randomization procedure**)? If yes, have they been described?	Yes	Materials and Methods
Include a statement about **blinding** even if no blinding was done.	Yes	Materials and Methods
Describe **inclusion/exclusion criteria** if samples or animals were excluded from the analysis. Were the criteria pre-established?<br>If sample or data points were omitted from analysis, report if this was due to attrition or intentional exclusion and provide justification.	Yes	Materials and Methods
For every figure, are **statistical tests** justified as appropriate? Do the data meet the assumptions of the tests (e.g., normal distribution)? Describe any methods used to assess it. Is there an estimate of variation within each group of data? Is the variance similar between the groups that are being statistically compared?	Yes	Materials and Methods, Figures

Sample definition and in-laboratory replication	Information included in the manuscript?	In which section is the information available? (Reagents and Tools Table, Materials and Methods, Figures, Data Availability Section)
In the figure legends: state number of times the experiment was **replicated** in laboratory.	Yes	Materials and Methods, Figures
In the figure legends: define whether data describe **technical or biological replicates**.	Yes	Materials and Methods, Figures

Ethics

Ethics	Information included in the manuscript?	In which section is the information available? (Reagents and Tools Table, Materials and Methods, Figures, Data Availability Section)
Studies involving **human participants**: State details of **authority granting ethics approval** (IRB or equivalent committee(s), provide reference number for approval.	Not Applicable	
Studies involving **human participants**: Include a statement confirming that **informed consent** was obtained from all subjects and that the experiments conformed to the principles set out in the WMA Declaration of Helsinki and the Department of Health and Human Services Belmont Report.	Yes	Materials and Methods
Studies involving **human participants: ** For publication of **patient photos**, include a statement confirming that consent to publish was obtained.	Not Applicable	
Studies involving experimental **animals**: State details of **authority granting ethics approval** (IRB or equivalent committee(s), provide reference number for approval. Include a statement of compliance with ethical regulations.	Yes	Materials and Methods
Studies involving **specimen and field samples:** State if relevant **permits** obtained, provide details of authority approving study; if none were required, explain why.	Not Applicable	

Dual Use Research of Concern (DURC)	Information included in the manuscript?	In which section is the information available? (Reagents and Tools Table, Materials and Methods, Figures, Data Availability Section)
Could your study fall under dual use research restrictions? Please check biosecurity documents and list of **select agents and toxins** (CDC): https://www.selectagents.gov/sat/list.htm	Not Applicable	
If you used a select agent, is the security level of the lab appropriate and reported in the manuscript?	Not Applicable	
If a study is subject to dual use research of concern regulations, is the name of the **authority granting approval and reference number** for the regulatory approval provided in the manuscript?	Not Applicable	

Reporting

The MDAR framework recommends adoption of discipline-specific guidelines, established and endorsed through community initiatives. Journals have their own policy about requiring specific guidelines and recommendations to complement MDAR.

Adherence to community standards	Information included in the manuscript?	In which section is the information available? (Reagents and Tools Table, Materials and Methods, Figures, Data Availability Section)
State if relevant guidelines or checklists (e.g., **ICMJE, MIBBI, ARRIVE, PRISMA**) have been followed or provided.	Yes	Materials and Methods
For **tumor marker prognostic studies,** we recommend that you follow the **REMARK** reporting guidelines (see link list at top right). See author guidelines, under 'Reporting Guidelines'. Please confirm you have followed these guidelines.	Not Applicable	
For **phase II and III randomized controlled trials**, please refer to the **CONSORT** flow diagram (see link list at top right) and submit the CONSORT checklist (see link list at top right) with your submission. See author guidelines, under 'Reporting Guidelines'. Please confirm you have submitted this list.	Not Applicable	

Data Availability

Data availability	Information included in the manuscript?	In which section is the information available? (Reagents and Tools Table, Materials and Methods, Figures, Data Availability Section)
Have **primary datasets** been deposited according to the journal's guidelines (see 'Data Deposition' section) and the respective accession numbers provided in the Data Availability Section?	Not Applicable	
Were **human clinical and genomic datasets** deposited in a public access-controlled repository in accordance to ethical obligations to the patients and to the applicable consent agreement?	Not Applicable	
Are **computational models** that are central and integral to a study available without restrictions in a machine-readable form? Were the relevant accession numbers or links provided?	Yes	Materials and Methods
If publicly available data were reused, provide the respective **data citations in the reference list**.	Not Applicable	
